# Update and taxonomic revision of *Ganoderma* (*Basidiomycota*, *Ganodermataceae*) from China

**DOI:** 10.3897/mycokeys.140.204190

**Published:** 2026-09-21

**Authors:** Han-Chi Lei, JIa Li, Tai-Jie Yu, Yu-Xian Gao, Zhong-Hua Wu, Yu-Cheng Dai, Tie-Zhi Liu, Run-Ke Mao, Wei-Jian Guan, Ying Tang, Ting Li, Yuan-Tian Lu, Qing-Wei Zang, Li-Ping Tang

**Affiliations:** 1 School of Pharmaceutical Sciences and Yunnan Key Laboratory of Pharmacology for Natural Products, Kunming Medical University, Kunming 650500, China School of Pharmaceutical Sciences and Yunnan Key Laboratory of Pharmacology for Natural Products, Kunming Medical University Kunming China; 2 Yunnan College of Modern Biomedical Industry, Kunming 650500, China Yunnan College of Modern Biomedical Industry Kunming China; 3 Lufeng City Ruicao Planting Farm, Chuxiong 651213, China Lufeng City Ruicao Planting Farm Chuxiong China; 4 Institute of Microbiology, School of Ecology and Nature Conservation, Beijing Forestry University, Beijing 100083, China Institute of Microbiology, School of Ecology and Nature Conservation, Beijing Forestry University Beijing China; 5 College of Chemistry and Life Sciences, Chifeng University, Chifeng 024000, China College of Chemistry and Life Sciences, Chifeng University Chifeng China; 6 Lizi Forest Farm of Xiaolongshan Forestry Protection Center in Gansu Province, Tianshui 741004, China Lizi Forest Farm of Xiaolongshan Forestry Protection Center in Gansu Province Tianshui China; 7 Xizang Guangyao Hanfang Ganoderma Co., Ltd., Linzhi 860300, China Xizang Guangyao Hanfang Ganoderma Co., Ltd. Linzhi China; 8 Yunnan Ruicao Health Industry Limited Liability Company, Kunming 650500, China Yunnan Ruicao Health Industry Limited Liability Company Kunming China; 9 Yunnan Mingyangtianxia Green Food Co., Ltd., Kunming 650500, China Yunnan Mingyangtianxia Green Food Co., Ltd. Kunming China

**Keywords:** Medicinal fungi, new record, new taxa, southwestern China, synonym

## Abstract

China possesses a particularly high diversity of *Ganoderma*, a genus of fungi with significant pharmacological relevance. This study provides an integrative taxonomic revision of Chinese *Ganoderma* species, utilizing a four-locus molecular dataset (ITS, nrLSU, *TEF1-α*, and *RPB2*) in conjunction with comprehensive morphological and ecological analyses. Two new species from southwestern China, *G. roseogelatum* (粉胶灵芝) **sp. nov**. and *G. sinolucidum* (中华亮盖灵芝) **sp. nov**., are described and illustrated. *G. sinolucidum* has previously been misidentified as the European *G. lucidum* throughout eastern and southeastern Asia. Our study clarifies that while true *G. lucidum* occurs in both Europe and temperate East Asia (e.g., northern China), the tropical to subtropical Asian collections previously referred to it represent the distinct new species *G. sinolucidum*, thereby resolving this long-standing misapplication. Furthermore, two species are newly reported for China: *G. eickeri* (艾氏灵芝), originally described from South Africa, is documented outside Africa for the first time, indicating a novel Afro-Asian disjunct distribution; and *G. oregonense* (俄勒冈灵芝), originally described from North America, is confirmed in Asia through sequencing of the *G. tibetanum* holotype. This finding establishes *G. oregonense* as the third *Ganoderma* species with a verified Asia-North America disjunct distribution, following *G. applanatum* and *G. tsugae*. Re-examination of type specimens leads to the synonymization of *G. weixiense* with *G. leucocontextum***syn. nov**., and *G. tibetanum* with *G. oregonense***syn. nov**. Sequencing the ITS region from the *G. tibetanum* holotype resolves longstanding taxonomic uncertainty and confirms the presence of *G. oregonense* in China. These results refine the taxonomic understanding of *Ganoderma* diversity in China and correct persistent misapplications of European names to Asian taxa. The study demonstrates the importance of integrating molecular phylogenetics with morphological reassessment to clarify species boundaries within this ecologically and pharmacologically significant genus.

## Introduction

The genus *Ganoderma* P. Karst. comprises wood-decomposing fungi with a global distribution. Morphologically, it is characterized by annual to perennial basidiomata, pilei ranging from dull brown to distinctly laccate, and brownish pore surfaces upon bruising; key microscopic features include double-walled, chambered basidiospores, and a trimitic hyphal system (Pristas et al. 2023). Ecologically, *Ganoderma* species serve as efficient wood-rotting agents, though some pathogenic species, such as *G. boninense* Pat., *G. philippii* (Bres. & Henn. ex Sacc.) Bres. and *G. zonatum* Murrill, cause basal stem rot in trees, leading to substantial economic losses (Utomo et al. 2005; Zhou and Dai 2012; Ho and Tan 2015; He et al. 2022; Yuan et al. 2023). Conversely, several species, most notably *G. lingzhi* Sheng H. Wu et al. and *G. sinense* J.D. Zhao et al., have been widely used in traditional medicine across Asian countries for treating sleep disorders, immune dysregulation, allergies, hypertension, and cancer, as well as for their antihypertensive, antioxidant, antidiabetic, hepatoprotective, immunomodulatory, neurogenic, and nephroprotective properties (Xu et al. 2009; Wu et al. 2019; El Sheikha 2022; Du et al. 2023; Rašeta et al. 2026). More recently, antiviral properties, including potential activity against COVID-19 have also been explored (Karunarathna et al. 2025). The transition of *Ganoderma* from traditional folk medicine to modern dietary supplement industries has attracted increasing attention (Karunarathna et al. 2025).

Despite its importance, the taxonomy of *Ganoderma* remains beset by several intertwined challenges. First, the high phenotypic plasticity of basidiomata indeterminate growth, color changes with age, and convergent morphological traits often renders morphology-based identification unreliable (Zhao and Zhang 2000). Second, the loss or poor preservation of many type specimens, compounded by the absence of molecular sequence data for historically described species, frequently obscures species boundaries. Third, while molecular systematics, particularly multi-locus phylogenetics, has become essential for resolving cryptic diversity within *Ganoderma* (Moncalvo et al. 1995; Yang et al. 2018), many previously reported species lack such evidence (Cui et al. 2023). Standardized cultivation practices for *Ganoderma* remain challenging due to taxonomic ambiguities, as different species may require different substrates and conditions (Karunarathna et al. 2025); consequently, both synonymy (different names for same species) and misidentification (the same name applied to different species) are rampant, hampering pharmacological reproducibility, cultivation, and conservation efforts, given that different *Ganoderma* species or even misidentified products, may exhibit distinct bioactive profiles (Ghafoor et al. 2025; Rašeta et al. 2026).

A paradigmatic example is the *G. lucidum* species complex. *Ganoderma lucidum* (Curtis) P. Karst., the type species of the genus, was originally described from Europe (Karsten 1881; Donk 1948). However, the name has been widely misapplied to morphologically similar laccate species worldwide (Xing et al. 2018; Luangharn et al. 2019; Sun et al. 2022). Moncalvo et al. (1995) and Moncalvo and Ryvarden (1997) suggested that true *G. lucidum* might be restricted to Europe, with many extralimital records representing distinct taxa; however, subsequent studies, including the present work, have confirmed its occurrence also in temperate East Asia. For instance, *G. tsugae* Murrill from the United States is confined to northern coniferous forests (Adaskaveg and Gilbertson 1986), while its specimens from Australia previously identified as “*G. lucidum*” were reassigned to *G. steyaertanum* B.J. Smith & K. Sivasithamparam (Smith and Sivasithamparam 2003). In Asia, the situation is equally complex. Wang et al. (2009) re-examined the widely cultivated “*G. lucidum*” in Taiwan, and identified *G. multipileum* Ding Hou, further proposing six synonyms for *G. orbiforme* Ryvarden. Subsequent studies have shown that the name “*G. lucidum*” has been applied to several Asian species, including *G. leucocontextum*, *G. lingzhi*, *G. sichuanense* J.D. Zhao & X.Q. Zhang, and *G. shanxiense* L. Fan & H. Liu (Cao et al. 2012; Li et al. 2014; Ye et al. 2019; Du et al. 2023; Liu et al. 2024). In a series of revisions, Chinese researchers proposed dozens of synonyms, underscoring the prevalence of nomenclatural redundancy within Chinese *Ganoderma* (Wang 2012; Cui et al. 2023). These persistent taxonomic ambiguities directly impede accurate species delimitation and the reliable evaluation of their medicinal properties.

China, with its vast territory and diverse climate zones, hosts a remarkably high diversity of *Ganoderma*. To date, 49 species have been reported, including *G. leucocontextum* T.H. Li et al. and *G. weixiense* Karun. & J.C. Xu, excluding taxonomically doubtful taxa such as *G. tibetanum* J.D. Zhao & X.Q. Zhang (Cao et al. 2012; Cui et al. 2023; He et al. 2023). Among them, *G. leucocontextum* and *G. weixiense* are particularly noteworthy. Both species are distributed in high-altitude regions of southwestern China and, according to their original descriptions, are morphologically highly similar, differing mainly in basidiospore size. Furthermore, both taxa were described solely on the basis of ITS sequences, lacking multi-locus phylogenetic evidence (Li et al. 2014; Ye et al. 2019). A critical re-examination of their type specimens is therefore necessary to determine whether these two names represent a single species. Similarly, the taxonomic status of *G. tibetanum* has never been critically reassessed with molecular evidence (Cui et al. 2023).

Southwestern China, especially Yunnan Province, has indeed yielded a considerable number of newly described *Ganoderma* species in recent years (Cui et al. 2023). Nevertheless, cryptic diversity remains to be fully resolved due to morphological plasticity and the frequent absence of multi-locus data for regional collections. This is particularly evident in several folk medicinal taxa that are locally cultivated and traded–such as “Gasa Lingzhi” from Xinping County and “Xue Lingzhi” from central Yunnan—but lack formal scientific names, posing risks for therapeutic reproducibility and cultivation standardization. An additional long-standing controversy concerns the geographic distribution of *G. lucidum*: although some authors have suggested that this species is restricted to Europe (Moncalvo and Ryvarden 1997), the name has been persistently applied to morphologically similar Asian collections, raising the question of whether this reflects a broader natural distribution or widespread misidentification.

To address these gaps, this study focuses on biodiversity-rich regions in China, including Anhui, Yunnan, and Xizang. By integrating morphological observations with four-locus (ITS, nrLSU, *TEF1-α*, and *RPB2*) phylogenetic analysis, we achieved the following: (1) described two new species: *G. roseogelatum* sp. nov. (the cultivated “Gasa” lingzhi) and *G. sinolucidum* sp. nov. (the cultivated “Xue Lingzhi”, which has been consistently misidentified as the European *G. lucidum* in China); (2) report two new Chinese records: *G. eickeri* Tchotet, M.P.A. Coetzee (originally described from South Africa) and *G. oregonense* Murrill (described from North America); and (3) clarify taxonomic ambiguities surrounding *G. weixiense* and *G. tibetanum* by proposing their synonymization under *G. leucocontextum* syn. nov. and *G. oregonense* syn. nov., respectively. These findings not only refine the taxonomic framework of *Ganoderma* diversity in China, but also resolve longstanding misapplications of a European name to Asian taxa. This study provides a reliable foundation for future medicinal applications, cultivation practices, and biodiversity conservation.

## Materials and methods

### Collection sites and sampling

Specimens were collected from the provinces of Anhui, Xizang, and Yunnan. Fresh basidiomata were photographed, tagged (Rathnayaka et al. 2025), and documented in the field, then dried at 40–45 °C for 12 hours. The newly collected specimens were deposited in the Mycological Herbarium of Kunming Medical University (MHKMU). In addition to these newly collected materials, we also borrowed and re-examined relevant type specimens and historical collections from the following herbaria for comparative morphological and molecular analyses: Mycological Herbarium of Chifeng University (CFSZ); Herbarium Mycologicum Academiae Sinicae (HMAS); Herbarium of Cryptogams, Kunming Institute of Botany, Chinese Academy of Sciences (HKAS); and Herbarium of Guangdong Institute of Microbiology (GDGM), Guangzhou. All borrowed specimens were carefully examined, and their voucher information is provided in the taxonomic section.

### Morphological studies

Macroscopic descriptions were based on detailed notes and photographs of fresh basidiomata. Color terminology followed Kornerup and Wanscher (1978).

Morphological analyses were conducted as instructed by Wang and Yao (2005) and Torres-Torres and Guzmán-Dávalos (2012). Microscopic examinations and anatomical features (basidia, basidiospores, hyphal system, and pileipellis) were observed and photographed using a Leica DM2500 light microscope. A 5% KOH solution and a 1% Congo Red solution (w/v) served as the mounting medium and stain, respectively.

The following abbreviations are used: IKI = Melzer’s reagent, IKI– = neither

amyloid nor dextrinoid, KOH = 5% potassium hydroxide, CB = Cotton blue, CB+ = cyanophilous (Xing et al. 2016). The notation [*n/m/p*] indicates that measurements were derived from “*n*” basidiospores measured from “*m*” basidiomata of “*p*” collections. Basidiospore dimensions are presented as (*a*)*b*–*c*(*d*), where “*a*” is the minimum, “*d*” the maximum, and “*b*–*c*” represents the range of 90% of the measured values. Additionally, *L* denotes the mean spore length, *W* the mean spore width, *Q* the length/width ratio of the basidiospores, and *Q_m_* denotes the average of *n* measured basidiospores ± standard deviation (He et al. 2021; Zheng et al. 2021).

In this study, the “Known distribution” for each species is restricted based on specimens examined with morphological and/or molecular confirmation. Records inferred exclusively from GenBank sequences without corresponding voucher re-examination are explicitly indicated as “phylogenetically inferred” and are not treated as confirmed distribution ranges unless stated otherwise.

### DNA extraction, amplification, and sequencing

DNA extraction and PCR amplification were performed as described by Tang et al. (2017). Four loci were amplified: internal transcribed spacer (ITS), nuclear large ribosomal subunit (nrLSU), the translation elongation factor 1-alpha (*TEF1-α*), and the second largest subunit of RNA polymerase II (*RPB2*). The primer pairs used were: ITS5/ITS4 for ITS, LR0R/LR5 for nrLSU, TEF1-983F/TEF1-1567R for *TEF1-α*, and RPB2-6F/fRPB2-7cR for *RPB2*.

Each 25 μL PCR mixture contained 12.5 μL of 2× Taq PCR Master Mix, 9.5 μL of ddH_2_O, 1 μL of each 10 μM primer, and 1 μL of DNA. The thermal cycling program for ITS was: 94 °C for 5 min, followed by 35 cycles of 94 °C for 40 s, 58 °C for 40 s, 72 °C for 1 min; and a final extension at 72 °C for 10 min. For nrLSU and *TEF1-α*: 94 °C for 5 min, followed by 35 cycles at 94 °C for 40 s, 54 °C for 40 s, and 72 °C for 1 min; final extension at 72 °C for 10 min. For *RPB2*, the same program was used except with an annealing temperature of 52 °C.

PCR products were verified by 1% (w/v) agarose gel electrophoresis, stained with ethidium bromide, and visualized under UV light. Products showing a bright single band were purified and sequenced using an ABI 3730xl DNA Analyzer with the same primers.

Raw sequences were analyzed using BioEdit software. Chromatograms were manually checked for degenerate bases, indels, sequencing errors, and double peaks. High-quality sequences were subjected to BLAST searches against GenBank for initial identification. Reference sequences of closely related species were retrieved from GenBank for phylogenetic analysis (Sun et al. 2022).

### Phylogenetic analyses

The combined nuclear dataset (ITS, nrLSU, *TEF1-α*, and *RPB2*) was analyzed using maximum likelihood (ML) and Bayesian inference (BI). Sequence alignment was performed with MAFFT v.7 (http://mafft.cbrc.jp/alignment/server/), followed by visual inspection and manual optimization using BioEdit v.7.0.9 to improve alignment accuracy and minimize gaps. The alignment was then converted to Phylip format for RAxML analysis. ML tree generation and bootstrap analysis were conducted using RAxML7.2.6 with 1,000 bootstrap replicates combined with an ML search. All model parameters were estimated by the software.

Bayesian inference was conducted using MrBayes v. 3.2.7. The analysis was run for 2,000,000 generations, with trees sampled every 1,000 generations. The run was stopped when the average standard deviation of split frequencies fell below 0.01, indicating convergence.

Both maximum likelihood (ML) and Bayesian inference (BI) analyses yielded largely congruent topologies; therefore, only the ML tree is shown. Bootstrap support (BS) values ≥ 70% and Bayesian posterior probabilities (PP) ≥ 0.95 are indicated above the nodes in the resulting phylogram.

Phylogenetic trees were visualized using FigTree v1.4.4, and figures were prepared for publication using Adobe Illustrator CS5.

## Results

### Molecular data

In this study, a total of 46 new sequences (18 ITS, 16 nrLSU, 9 *TEF1-α*, and 3 *RPB2*) were generated from 18 specimens and have been deposited in GenBank (Table 1). The final concatenated dataset comprised 2,559 aligned sites, including gaps, distributed as follows: ITS: 1–741; nrLSU: 742–2,076; *TEF1-α*: 2,077–3,162; *RPB2*: 3,163–4,258. Following previous studies (Sun et al. 2020, 2022; Luangharn et al. 2021), *Amauroderma rugosum* (Blume & T. Nees) Torrend and *Sanguinoderma rude* (Berk.) Y.F. Sun, D.H. Costa & B.K. Cui were selected as outgroups. The estimated base frequencies were as follows: A = 0.234, C = 0.248, G = 0.273, T = 0.245; substitution rates were AC = 1.05315, AG = 4.87048, AT = 1.07539, CG = 1.41975, CT = 8.59571, GT = 1.00000; the shape parameter (α) of the gamma distribution was 0.416, and the tree length was 1.350.

**Table 1.** Taxa names and their corresponding information including GenBank accession numbers used in the phylogenetic analyses.

**Species**	**Taxonomic authority**	**Voucher no**.	**Locality**	**GenBank accession No**.				**References**
				**ITS**	**LSU**	***TEF1*** *-* ***α***	***RPB2***	
*Amauroderma rugosum*	(Blume & T. Nees) Torrend	Cui9011	China	KJ531664	KU220010	KU572504	MK121517	Li et al. 2014
*Ganoderma adspersum*	(Schulzer) Donk	ACAM2012-0050	Unknown	MG706203	MG706153	MG837827	MG837779	GenBank
*G. adspersum*	(Schulzer) Donk	ACAM-A113	Unknown	MG706204	MG706154	—	—	GenBank
*G. applanatum*	(Pers.) Pat.	Cui14062	Jilin, China	MZ354913	MZ355076	MZ221635	MZ358846	Sun et al. 2022
*G. applanatum*	(Pers.) Pat.	Cui14070	Jilin, China	MZ354914	MZ355079	MZ221636	MZ245387	Sun et al. 2022
*G. bubalinomarginatum*	B.K. Cui, J.H. Xing & Y.F. Sun	Dai20074	Guangxi, China	MZ354926	MZ355010	MZ221637	MZ245388	Sun et al. 2022
*G. bubalinomarginatum*	B.K. Cui, J.H. Xing & Y.F. Sun	BJFC031749 (T)	Guangxi, China	NR_182905	—	—	—	Sun et al. 2022
*G. calidophilum*	J.D. Zhao, L.W. Hsu & X.Q. Zhang	MFLU19-2219	China	MN398338	—	—	—	Luangharn et al. 2019
*G. calidophilum*	J.D. Zhao, L.W. Hsu & X.Q. Zhang	MFLU19-2174	China	MN398337	—	—	—	Luangharn et al. 2019
*G. carnosum*	Pat.	XC23-147	France	PV121509	PV121818	—	—	GenBank
*G. carnosum*	Pat.	ZV012PN	Slovakia	MK415314	MK995638	MN017812		GenBank
*G. carocalcareum*	Douanla-Meli & Langer	DMC513	Cameroon	EU089970	—	—	—	Douanla-Meli and Langer 2009
*G. carocalcareum*	Douanla-Meli & Langer	DMC322 (T)	Cameroon	EU089969	—	—	—	Douanla-Meli and Langer 2009
*G. casuarinicola*	J.H. Xing, B.K. Cui & Y.C. Dai	Dai16336 (T)	Guangdong, China	NR_158432	MZ355103	MG367565	MG367508	Xing et al. 2018
*G. casuarinicola*	J.H. Xing, B.K. Cui & Y.C. Dai	HKAS104639	Thailand	MK817650	MK817654	—	MK840868	Luangharn et al. 2019
*G. curtisii*	Berk.	CBS100131	USA	JQ781848	—	KJ143926	KJ143966	Cao et al. 2012
*G. curtisii*	Berk.	CBS100132	USA	JQ781849	—	KJ143927	KJ143967	Cao et al. 2012
*G. dianzhongense*	J. He & S.H. Li	HKAS110005 (T)	Yunnan, China	NR_177159	—	MW838993	—	He et al. 2022
*G. dianzhongense*	J. He & S.H. Li	HKAS112719	Yunnan, China	—	—	MW838996	MW839002	He et al. 2022
*G. dunense*	Tchotet, M.P.A. Coetzee, Rajchenb. & Jol. Roux	CMW42157 (T)	South Africa	MG020255	—	MG020227	—	Tchotet-Tchoumi et al. 2018
*G. dunense*	Tchotet, M.P.A. Coetzee, Rajchenb. & Jol. Roux	CMW42149	South Africa	MG020248	—	MG020226	—	Tchotet-Tchoumi et al. 2018
*G. ecuadoriense*	Steyaert	URM89449	Brazil	MK119828	MK119908	MK121577	MK121535	Sun et al. 2022
*G. ecuadoriense*	Steyaert	Dai17397	Brazil	MZ354950	MZ355019	MZ221648	MZ245398	Sun et al. 2022
*G. eickeri*	Tchotet, M.P.A. Coetzee, Rajchenb. & Jol. Roux	CMW49692 (T)	South Africa	MH571690	—	MH567287	—	Tchotet-Tchoumi et al. 2019
*G. eickeri*	Tchotet, M.P.A. Coetzee, Rajchenb. & Jol. Roux	Dai12595	South Africa	MZ354964	MZ355035	MZ221651	—	Sun et al. 2022
***G. eickeri***	Tchotet, M.P.A. Coetzee, Rajchenb. & Jol. Roux	**MHKMU GYX-04**	**Anhui, China**	**PX611870**	**PV446184**	—	—	**This study**
*“G. eickeri”* (= *G. gibbosum*)	= *G. gibbosum* (Blume & T. Nees) Pat.	GXU2692	Guangxi, China	PP845228	PP847213	—	PP960231	Wei et al. 2024
*G. ellipsoideum*	K.K. Hapuar. & T.C. Wen	GACP14080966 (T)	Hainan, China	NR_160617	—	—	—	Hapuarachchi et al. 2018
*G. ellipsoideum*	K.K. Hapuar. & T.C. Wen	Dai19683	Yunnan, China	MZ354970	MZ355018	MZ221653	—	Sun et al. 2022
*G. enigmaticum*	M.P.A. Coetzee, Marinc., Muthelo & M.J. Wingf.	CBS139792 (T)	South Africa	NR_132918	NG_058156	—	—	Coetzee et al. 2015
*G. flexipes*	Pat.	Cui13863	Hainan, China	MZ354924	MZ355064	MZ221656	MZ245402	Sun et al. 2022
*G. flexipes*	Pat.	Cui13841	Hainan, China	MZ354923	MZ355063	MZ221655	MZ245401	Sun et al. 2022
*G. gibbosum*	(Blume & T. Nees) Pat.	HKAS97411	China	MN398341	MN428666	—	—	Luangharn et al. 2019
*G. gibbosum*	(Blume & T. Nees) Pat.	Cui17254	Sichuan, China	MZ354966	MZ355115	MZ221660	MZ245406	Sun et al. 2022
*G. hochiminhense*	Luangharn, Karun. & K.D. Hyde	MFLU19-2225	Vietnam	MN396662	MN396391	MN423177	—	Luangharn et al. 2019
*G. hochiminhense*	Luangharn, Karun. & K.D. Hyde	MFLU19-2224 (T)	Vietnam	MN398324	MN396390	MN423176	—	Luangharn et al. 2019
*G. leucocontextum*	T.H. Li, W.Q. Deng, Sheng H. Wu, Dong M. Wang & H.P. Hu	GDGM40200 (T)	Xizang, China	NR_154902	—	—	—	Li et al. 2014
*G. leucocontextum*	T.H. Li, W.Q. Deng, Sheng H. Wu, Dong M. Wang & H.P. Hu	GDGM42932	Xizang, China	KF372586	—	—	—	Li et al. 2014
***G. leucocontextum***	**T.H. Li, W.Q. Deng, Sheng H. Wu, Dong M. Wang & H.P. Hu**	**MHKMU PYJ-386**	**Yunnan, China**	**PV446174**	**PV446190**	**PX026327**	**PX069023**	**This study**
***G. leucocontextum***	**T.H. Li, W.Q. Deng, Sheng H. Wu, Dong M. Wang & H.P. Hu**	**MHKMU PYJ-037**	**Yunnan, China**	**PV446170**	**PV446186**			**This study**
***G. leucocontextum***	**T.H. Li, W.Q. Deng, Sheng H. Wu, Dong M. Wang & H.P. Hu**	**MHKMU ZWH-078**	**Yunnan, China**	**PV446171**	**PV446187**	—	—	**This study**
***G. leucocontextum***	**T.H. Li, W.Q. Deng, Sheng H. Wu, Dong M. Wang & H.P. Hu**	**MHKMU ZWH-321**	**Yunnan, China**	**PV446173**	**PV446189**	**PX026326**	**PX069022**	**This study**
***G. leucocontextum***	**T.H. Li, W.Q. Deng, Sheng H. Wu, Dong M. Wang & H.P. Hu**	**MHKMU LHC-133**	**Xizang, China**	**PV446172**	**PV446188**	—	—	**This study**
*G. lingzhi*	Sheng H. Wu, Y. Cao & Y.C. Dai	Wu1006-38 (T)	Hubei, China	JQ781858	—	JX029976	JX029980	Cao et al. 2012
*G. lingzhi*	Sheng H. Wu, Y. Cao & Y.C. Dai	Dai20895	China	MZ354904	MZ355006	MZ221668	MZ245413	Sun et al. 2022
*G. lucidum*	(Curtis) P. Karst.	MUCL31549	France	MK554777	MG706176	MG837845	MG837804	Cabarroi-Hernández et al. 2019
*G. lucidum*	(Curtis) P. Karst.	LGAM504 - ACAM2013-0062	Greece	MG706229	MG706175	MG837844	MG837803	GenBank
***G. lucidum***	**(Curtis) P. Karst**.	**MHKMU MM -343**	**Jilin, China**	**PV446162**	**PV446179**	—	—	**This study**
***G. lucidum***	**(Curtis) P. Karst**.	**CFSZ 18739**	**Inner Mongolia, China**	**PX611871**	—	—	—	**This study**
***G. lucidum***	**(Curtis) P. Karst**.	**CFSZ 21781**	**Inner Mongolia, China**	**PZ538873**	—	—	—	**This study**
*“G. lucidum”* (= *G. sinolucidum*)	= *G. sinolucidum* L.P. Tang, H.C. Lei, J. Li & Z.H. Wu, sp. nov.	Cui14404	Sichuan, China	MG279181	MZ355051	MG367573	MG367519	Xing et al. 2018
*“G. lucidum”* (= *G. sinolucidum*)	= *G. sinolucidum* L.P. Tang, H.C. Lei, J. Li & Z.H. Wu, sp. nov.	MFLU19-2162	Thailand	MN396341	—	—	MN423138	Luangharn et al. 2019
*G. mirabile*	(Lloyd) C.J. Humphrey	Cui18237	Malaysia	MZ354960	MZ355068	MZ221674	MZ345731	Sun et al. 2022
*G. mirabile*	(Lloyd) C.J. Humphrey	Cui18271	Malaysia	MZ354958	MZ355067	MZ221672	MZ345729	Sun et al. 2022
*G. multipileum*	Ding Hou	TWS31Auf (T)	Taiwan, China	MN533795	MN582953	MN621538	—	GenBank
*G. multipileum*	Ding Hou	Cui13597	China	MZ354899	MZ355043	MZ221675	MZ345732	Sun et al. 2022
*G. multiplicatum*	(Mont.) Pat.	SPC9	Brazil	KU569553	KU570951	—	—	Bolaños et al. 2016
*G. multiplicatum*	(Mont.) Pat.	ICN200402	Brazil	MW541360	MW546321	—	—	GenBank
*G. mutabile*	Y. Cao & S.H. Wu	Yuan2289 (T)	Yunnan, China	JN383977	—	—	—	Cao et al. 2012
*G. mutabile*	Y. Cao & S.H. Wu	Dai20414	Yunnan, China	MZ354977	MZ355110	MZ221680	MZ345735	Sun et al. 2022
*G. nasalanense*	K.K. Hapuar. & T.C. Wen	GACP17060211 (T)	Laos	NG_067696	—	—	—	Hapuarachchi et al. 2018
*G. nasalanense*	K.K. Hapuar. & T.C. Wen	LPDR17060212	Laos	MK345442	MK346832	—	—	Hapuarachchi et al. 2018
*G. obscuratum*	J. He & S.H. Li	HKAS123786 (T)	Yunnan, China	ON994237	OP456493	—	—	GenBank
*G. obscuratum*	J. He & S.H. Li	HKAS123772	Yunnan, China	ON994238	OP456494	—	—	GenBank
*G. orbiforme*	(Fr.) Ryvarden	Cui13891	China	MZ354953	MZ355017	MZ221682	MZ345736	Sun et al. 2022
*G. orbiforme*	(Fr.) Ryvarden	Cui18301	Malaysia	MZ354954	MZ355070	MZ221683	—	Sun et al. 2022
*G. oregonense*	Murrill	OMDL iNat # 238720255	USA	PX576822	—	—	—	GenBank
*G. oregonense*	Murrill	JLF1614	USA	MH277958	—	—	—	GenBank
*G. ovisporum*	H.D. Yang, Y. Ding & T.C. Wen	HKAS123193 (T)	Guizhou, China	NR_182704	MZ519545	—	MZ547661	Yang et al. 2022
*G. ovisporum*	H.D. Yang, Y. Ding & T.C. Wen	GACP20071602	China	MZ519548	MZ519546	—	MZ547662	Yang et al. 2022
*G. philippii*	(Bres. & Henn. ex Sacc.) Bres.	MFLU19-2223	Thailand	MN401411	MN398327	MN423175	—	Luangharn et al. 2019
*G. philippii*	(Bres. & Henn. ex Sacc.) Bres.	Cui14443	Hainan, China	MG279188	MZ355023	MG367578	MG367524	Xing et al. 2018
*G. platense*	Speg.	GM131	Uruguay	MN191597	—	—	—	Morera et al. 2021
*G. platense*	Speg.	NC5104	Uruguay	MN191589	—	—	—	Morera et al. 2021
*G. polychromum*	(Copel.) Murrill	330OR	USA	MG654196	—	MG754742	—	Loyd et al. 2018
*G. polychromum*	(Copel.) Murrill	CAB358	USA	OR736136	—	PP315948	OR853736	GenBank
*G. ravenelii*	Berk. & M.A. Curtis	MS187FL	USA	MG654211	—	MG754745	MG754865	Loyd et al. 2018
*G. ravenelii*	Berk. & M.A. Curtis	UMNFL153	USA	MG654210	—	—	—	Loyd et al. 2018
*G. resinaceum*	Boud.	MUCL52253	France	MK554786	—	MK554737	MK554764	Cabarroi-Hernández et al. 2019
*G. resinaceum*	Boud.	MUCL51491	Belgium	MK554775	—	MK554733	—	Cabarroi-Hernández et al. 2019
***G. roseogelatum***	**L.P. Tang, H.C. Lei, J. Li & Z.H. Wu, sp. nov**.	**MHKMU LHC-130**	**Yunnan, China**	**PV446164**	**PV446181**	**PX026322**	—	**This study**
***G. roseogelatum***	**L.P. Tang, H.C. Lei, J. Li & Z.H. Wu, sp. nov**.	**MHKMU LHC-137**	**Yunnan, China**	**PV446167**	—	**PX026324**	—	**This study**
***G. roseogelatum***	**L.P. Tang, H.C. Lei, J. Li & Z.H. Wu, sp. nov**.	**MHKMU LHC-184 (T)**	**Yunnan, China**	**PV446165**	**PV446182**	—	—	**This study**
***G. roseogelatum***	**L.P. Tang, H.C. Lei, J. Li & Z.H. Wu, sp. nov**.	**MHKMU LHC-185**	**Yunnan, China**	**PV446168**	—	—	—	**This study**
*G. sanduense*	K.K. Hapuar. & T.C. Wen	GACP18012501 (T)	Guizhou, China	NR_164049	—	—	MZ547663	Hapuarachchi et al. 2018
*G. sanduense*	K.K. Hapuar. & T.C. Wen	SA18012501	Guizhou, China	MK345450	—	—	—	Hapuarachchi et al. 2018
*G. sessile*	Murrill	111TX	USA	MG654306	—	MG754747	MG754866	Loyd et al. 2018
*G. sessile*	Murrill	JV 1209/27	USA	KF605630	—	KJ143937	KJ143976	GenBank
*“G. sessile”* (= *G. roseogelatum*)	= *G. roseogelatum* L.P. Tang, H.C. Lei, J. Li & Z.H. Wu, sp. nov.	NIFoS-XIIG-0441	South Korea	LC851590	—	—	—	Genbank
*G. shanxiense*	L. Fan & H. Liu	BJTC-FM423 (T)	Shanxi China	NR_172766	—	MK783937	MK783940	Liu et al. 2019
*G. shanxiense*	L. Fan & H. Liu	Dai18921	Yunnan, China	MZ354909	MZ355044	MZ221691	MZ345740	Sun et al. 2022
*G. sinense*	J.D. Zhao, L.W. Hsu & X.Q. Zhang	Wei5327	China	KF494998	KF495008	KF494976	MG367529	Xing et al. 2018
*G. sinense*	J.D. Zhao, L.W. Hsu & X.Q. Zhang	Dai20079	Hainan, China	MZ354962	MZ355058	MZ221696	MZ345745	Sun et al. 2022
***G. sinolucidum***	**L.P. Tang, H.C. Lei, J. Li & Z.H. Wu, sp. nov**.	**MHKMU MJ-06 (T)**	**Yunnan, China**	**PV446158**	**PV446175**	**PX026318**	**PX069021**	**This study**
***G. sinolucidum***	**L.P. Tang, H.C. Lei, J. Li & Z.H. Wu, sp. nov**.	**MHKMU LHC-127**	**Yunnan, China**	**PV446166**	**PV446183**	**PX026323**	—	**This study**
***G. sinolucidum***	**L.P. Tang, H.C. Lei, J. Li & Z.H. Wu, sp. nov**.	**MHKMU MM-487**	**Yunnan, China**	**PV446163**	**PV446180**	**PX026321**	—	**This study**
*G. suae*	J. He & S.H. Li	KUN-HKAS123775	Yunnan, China	PP869244	PP869251	—	—	He et al. 2024
*G. suae*	J. He & S.H. Li	KUN-HKAS123776 (T)	Yunnan, China	PP869243	PP869250	—	—	He et al. 2024
*G. subangustisporum*	J.H. Xing, B.K. Cui & Y.C. Dai	BJFC035453 (T)	Yunnan, China	NR_182909	NG_243700	—	—	Sun et al. 2022
*G. subangustisporum*	J.H. Xing, B.K. Cui & Y.C. Dai	Cui18597	Yunnan, China	MZ354980	MZ355025	MZ221700	MZ345746	Sun et al. 2022
*G. thailandicum*	Luangharn, Karun. & K.D. Hyde	HKAS104640 (T)	Thailand	MK848681	MK849879	—	—	Luangharn et al. 2019
*G. thailandicum*	Luangharn, Karun. & K.D. Hyde	HKAS104641	Thailand	MK848682	MK849880	—	—	Luangharn et al. 2019
***“G. tibetanum”* (= *G. oregonense*)**	**= *G. oregonense Murrill***	**HMAS 41261 (T)**	**Xizang, China**	**PZ537036**	**—**	**—**	**—**	**This study**
*G. tropicum*	(Jungh.) Bres.	Dai16434	Hainan, China	MG279194	MZ355026	MG367585	MG367532	Xing et al. 2018
*G. tropicum*	(Jungh.) Bres.	Dai19679	Guangdong, China	MZ354900	MZ355009	MZ221707	MZ358825	Sun et al. 2022
*G. tsugae*	Murrill	AFTOL-ID-771	Unknown	DQ206985	—	DQ059048	DQ408116	Matheny et al. 2007
*G. tsugae*	Murrill	UMNWI23	USA	MG654345	—	MG754766	—	Loyd et al. 2018
*“G. weixiense”* (= *G. leucocontextum*)	= *G. leucocontextum* T.H. Li, W.Q. Deng, Sheng H. Wu, Dong M. Wang & H.P. Hu	HKAS100649 (T)	Yunnan, China	NR_166271	NG_067863	MK302442	—	Ye et al. 2019
*G. wiiroense*	Douanla-Meli	MIN938704 (T)	Ghana	NR_158480	NG_064392	—	—	Genbank
*G. wiiroense*	Douanla-Meli	UMN-21-GHA	Ghana	KT952363	KT952364	—	—	GenBank
*G. williamsianum*	Murrill	Dai17790	Singapore	MZ354947	MZ355060	MZ221715	—	Sun et al. 2022
*G. williamsianum*	Murrill	Dai16809	Thailand	MG279183	MZ355030	MG367588	MG367535	Sun et al. 2022
*G. yunlingense*	B.K. Cui & Y.F. Sun	BJFC029587 (T)	Yunnan, China	NR_182902	NG_148997	—	—	Sun et al. 2022
*G. yunlingense*	B.K. Cui & Y.F. Sun	Cui16288	Yunnan, China	MZ354915	MZ355077	MZ221718	—	Sun et al. 2022
*G. yunnanense*	J. He & S.H. Li	HKAS123769	Yunnan, China	ON994236	OP373193	—	—	He et al. 2022
*G. yunnanense*	J. He & S.H. Li	HKAS123771 (T)	Yunnan, China	ON994235	OP373192	—	—	He et al. 2022
*Ganoderma* sp.	—	MKL25122	China	PX904627	—	PX907441	PX907440	GenBank
*Ganoderma* sp.	—	MUCL:46912	China	MK554791	—	MK554734	MK554758	Cabarroi-Hernández et al. 2019
*Sanguinoderma rude*	(Berk.) Y.F. Sun, D.H. Costa & B.K. Cui	Cui16592	Australia	MK119836	MK119916	MK121586	MK121521	Sun et al. 2022

Note: “—” indicates missing sequence data; “(T)” denotes type specimens; bold entries indicate specimen newly obtained in this study.

For each gene partition (ITS, nrLSU, *TEF1-α*, and *RPB2*), the optimal nucleotide substitution model was selected using ModelFinder based on the Bayesian Information Criterion (BIC). The best partitioning scheme resulted in two partitions: ITS, *TEF1-α*, and *RPB2* were combined into one partition under the TIM2+I+G4 model, whereas nrLSU was treated separately under the GTR+I+G4 model.

To assess potential conflicts among individual gene trees, we first reconstructed single-locus maximum likelihood (ML) trees with 1,000 bootstrap replicates. Topologies of the four single-locus trees were visually compared to identify well-supported nodes (bootstrap ≥ 70%) that were incongruent across loci. Only minor conflicts were observed among poorly supported nodes, which justified the use of the concatenation approach. For taxa with incomplete locus coverage (e.g., the Chinese collection of *G. eickeri* and the holotype of *G. tibetanum*, for which certain genes failed to amplify), their phylogenetic positions were primarily determined by the available overlapping loci. However, given their high ITS sequence similarity to respective type materials and congruent morphological traits, their placement in the concatenated tree is considered robust and is interpreted with appropriate caution.

**Figure 1.**
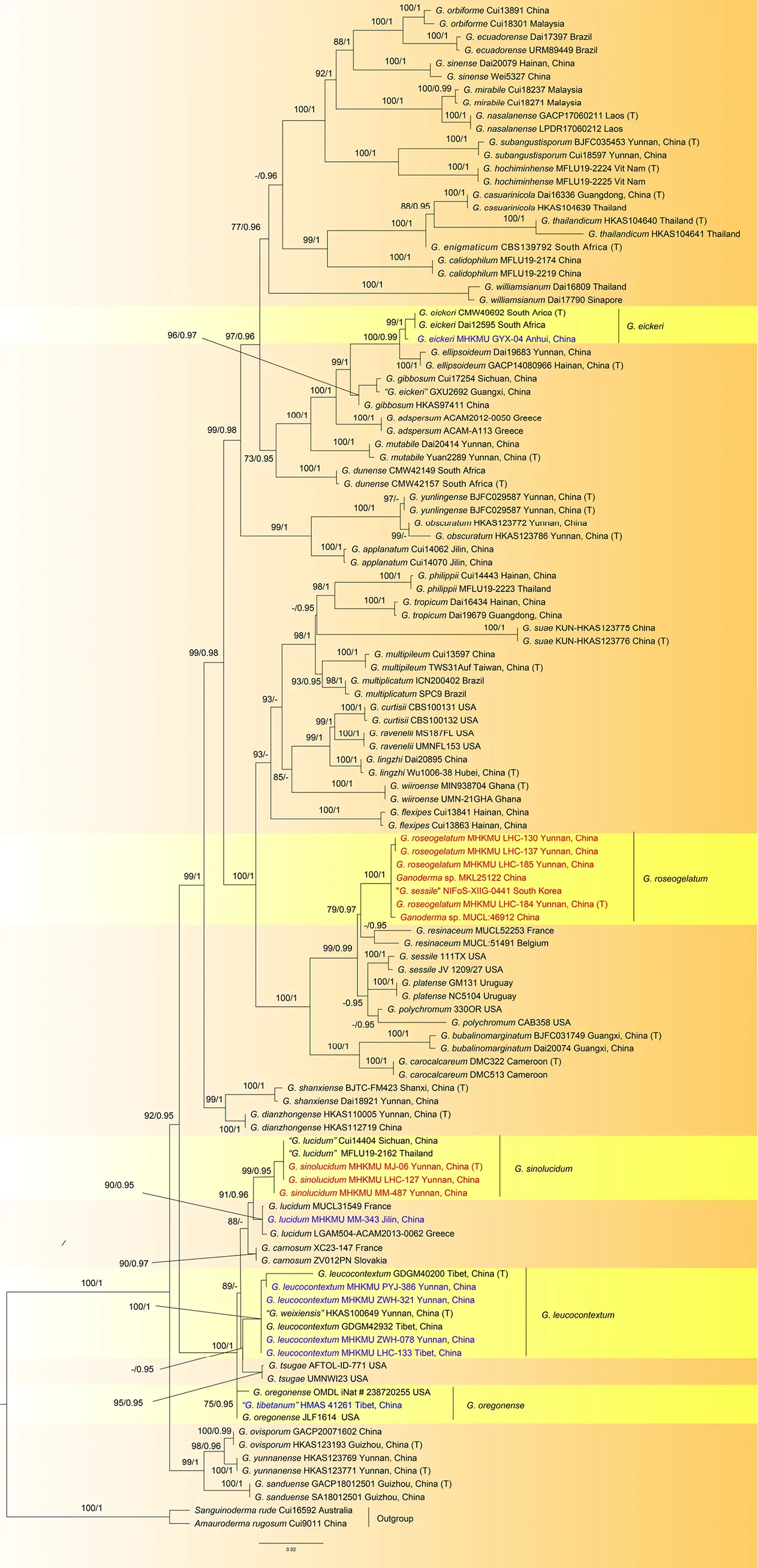
Phylogenetic tree of the genus *Ganoderma* based on the four-locus dataset (ITS, nrLSU, *TEF1-α*, and *RPB2*). RAxML BS values (≥70%) and Bayesian posterior probabilities (≥0.95) are shown above branches. Newly generated sequences are in color, and new species are in red. *Amauroderma rugosum* and *Sanguinoderma rude* were selected as outgroups (indicated at the base of the tree).

The multigene phylogenetic analyses showed that 18 Chinese specimens all fell within the genus *Ganoderma*. The four-locus phylogeny (Fig. 1) resolved the newly generated sequences into six terminal clades: *Ganoderma leucocontextum*, *G. lucidum*, *G. eickeri*, *G. oregonense*, and two distinct independent clades, which are proposed here as new species, *G. roseogelatum* sp. nov. and *G. sinolucidum* sp. nov. (BS = 100%, PP = 1.00; BS = 99%, PP = 0.95).

The clade of *G. roseogelatum* comprises four of our newly collected specimens and three additional sequences from GenBank, forming a strongly supported independent clade (BS = 100%, PP = 1.00), sister to *G. resinaceum* (BS = 94%, PP = 0.99).

The clade of *G. sinolucidum* includes three newly sampled specimens from Yunnan Province together with two sequences previously labeled as “*G. lucidum*”, forming a highly supported clade (BS = 99%, PP = 0.95), sister to *G. lucidum* (BS = 99%, PP = 0.96).

The newly recorded *G. eickeri* is represented by a Chinese collection (MHKMU GYX-04), which clusters with the African holotype (CMW 49692) in a strongly supported clade (BS = 100%, PP = 1.00). Notably, the sequences from the type specimens of *G. leucocontextum* and *G. weixiense* clustered together with strong support (BS = 100%, PP = 1.00), and the sequence from the holotype of *G. tibetanum* clustered with *G. oregonense* (BS = 75%, PP = 0.95), which suggests that each pair represents conspecific taxa.

### Taxonomy

#### Ganoderma eickeri

Taxon classificationFungiPolyporalesPolyporaceae

Tchotet, M.P.A. Coetzee, Rajchenb. & Jol. Roux, Mycologia 111(5): 730–747 (2019)

C101E241-B078-571D-BBED-B238BDA9D159

Fig. 2 A–H

##### Chinese name.

艾氏灵芝.

**Figure 2.**
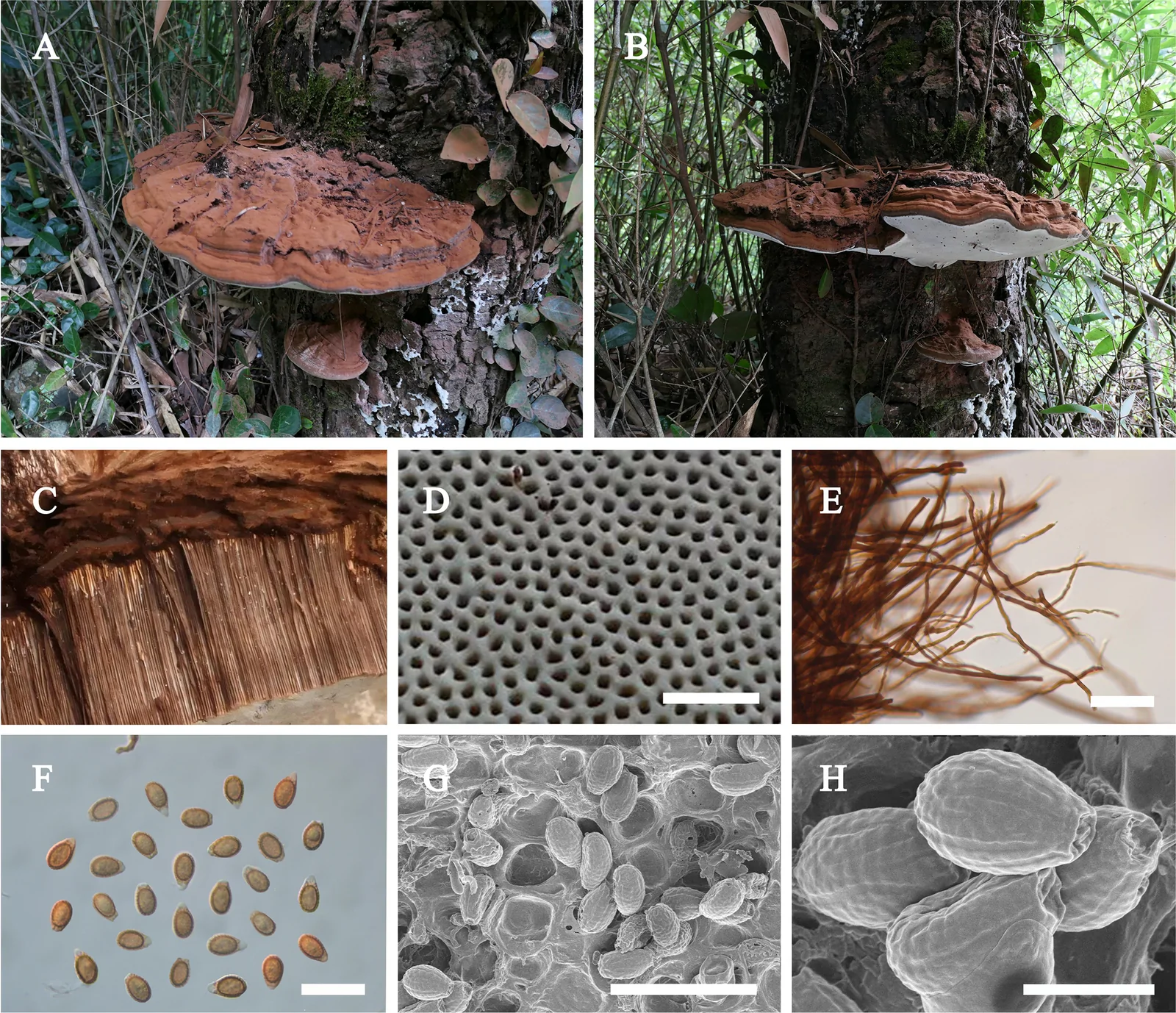
Macroscopic and microscopic features of *Ganoderma eickeri* (MHKMU GYX-04). **A, B**. Basidiomata; **C**. Cut side of pileus; **D**. Pore surface; **E**. Hyphae from context; **F**. Basidiospores; **G, H**. Basidiospores (SEM). Scale bars: 1 mm (**D**); 20 μm (**E, G**); 50 μm (**F**); 5 μm (**H**).

Based on the protologue (Tchotet-Tchoumi et al. 2019) and newly collected Chinese specimens.

##### Description.

***Basidiomata*** perennial, sessile, broadly attached, hard and corky. ***Pileus*** up to 33 × 12 cm and 5 cm thick at the base, solitary, circular to semicircular. *Pileus surface* red-brown, dull, smooth with faint radial cracks, often covered with dark brown spore deposits, surface concentrically zonate or azonate; margin obtuse, layered, slightly wavy. ***Context*** 4 mm thick, homogeneous, chocolate brown, lacking black melanoid lines. ***Tube*** up to 3.5 cm long, concolorous with context, stratified, corky. ***Pores*** 3–5 per mm (0.08–0.17 mm in diam.), predominantly triangular to quadrilateral, entire; dissepiments well developed, 0.05–0.18 mm thick; pore surface white when fresh, turning grayish white upon injury. ***Spore deposit*** dark red-brown.

***Hyphal system*: *Generative hyphae*** 2–4 μm in diam., thin-walled, with clamped connections, branched; ***Skeletal hyphae*** 4 μm in diam., hyaline to brown, thick-walled; IKI–, CB+; tissues darkening in KOH; ***Binding hyphae*** 3–4 µm in diam., light brown, with arboriform hyphae 1–2 µm in diam., flagelliform to dendritically branched, thick-walled, with hyaline walls.

***Basidiospores***: [20/1/1] (8.5–) 9.0–11.7(–11.9) × (5.5–)5.6–6.7 μm, *L* = 10.33 μm, *W* = 6.18 μm, *Q* = (1.51–)1.53–1.82(–1.93) (excluding myxosporium), *Q_m_* = 1.67 ± 0.12; ovoid to ellipsoid, with a conspicuously truncated apex, yellowish brown, inamyloid (IKI–); cyanophilous (CB+), double-walled, with moderately thick walls; under light microscopy, the exospore smooth, and the endospore walls bearing interwall pillars; under scanning electron microscopy, the exospore wall tuberculate, with ornamentation composed of numerous tubercles, typically isolated tubercles are longitudinally arranged into low ridges. ***Basidia*** not observed.

##### Specimens examined.

**China** • Anhui Province: Shitai County (石台县), Gao Lu Ting Village (高路亭村), 30°17'44"N, 117°46'41"E, elev. 370 m, 16 July 2024, on dead wood of *Pinus massoniana*, *Y. X. Gao* (MHKMU GYX-04).

##### Known distribution.

Africa—South Africa (Tchotet-Tchoumi et al. 2019). Asia—China, eastern region (Anhui province, at elevations of 300–400 m).

##### Notes.

*Ganoderma eickeri* was originally described from South Africa (Tchotet-Tchoumi et al. 2019). This study represents the first record of this species in China and the first report outside Africa. The Chinese collections (MHKMU GYX-04) are morphologically consistent with the type material in all diagnostic features summarized as follows: perennial, sessile, and broadly attached basidiomata (though larger, up to 33 cm vs. 8–12.5 cm in diam.); red-brown, dull, sulcate to zonate pileus surface with a thin crust; chocolate-brown, homogeneous context; stratified tube layer; pore surface white when fresh, turning grayish upon injury; pores 3–5 per mm (slightly more variable than 4–5 per mm in the type), triangular to quadrangular, with well-developed dissepiments; a trimitic hyphal system with clamped generative hyphae; and basidiospores ovoid to ellipsoid with a truncated apex, double-walled, and tuberculate under SEM, measuring as described above. The only notable difference is the larger basidiomata of the Chinese specimens (33 cm vs. 8–12.5 cm in diam.), which falls within the expected intraspecific variation for perennial polypores. Phylogenetically, the Chinese collections differ by only two bases from the type collection of *G. eickeri* (CMW 49692) in the ITS region. forming a strongly supported clade (BS = 100%) in our analysis. Although multi-locus data (TEF1-α and RPB2) are unavailable for the Chinese specimen due to DNA degradation, the combination of near-identical ITS sequences, full morphological concordance, and a well-supported phylogenetic placement provides sufficient evidence to confidently assign these collections to *G. eickeri*. This species represents the second *Ganoderma* taxon with molecular evidence supporting an Afro-Asian transcontinental distribution, following *G. orbiforme* (Fr.) Ryvarden originally from Guinea (Fries 1838; Ryvarden 2000; Hapuarachchi et al. 2019; Konara et al. 2025).

A Chinese sequence from Jiangxi Province (GenBank accession PP845228) was labeled as *G. eickeri*. However, our phylogenetic analysis places this sequence within the *G. gibbosum* clade, suggesting a likely misidentification.

#### Ganoderma leucocontextum

Taxon classificationFungiPolyporalesPolyporaceae

T.H. Li, W.Q. Deng, Sheng H. Wu, Dong M. Wang & H.P. Hu, Mycoscience 56 (1): 81–85 (2014) syn. nov.

ADB4A304-6F12-5CD9-B664-ECFAC562672B

Fig. 3A–O

##### Synonym.

*Ganoderma weixiense* Karun. & J.C. Xu (as “*G. weixiensis*”)

**Figure 3.**
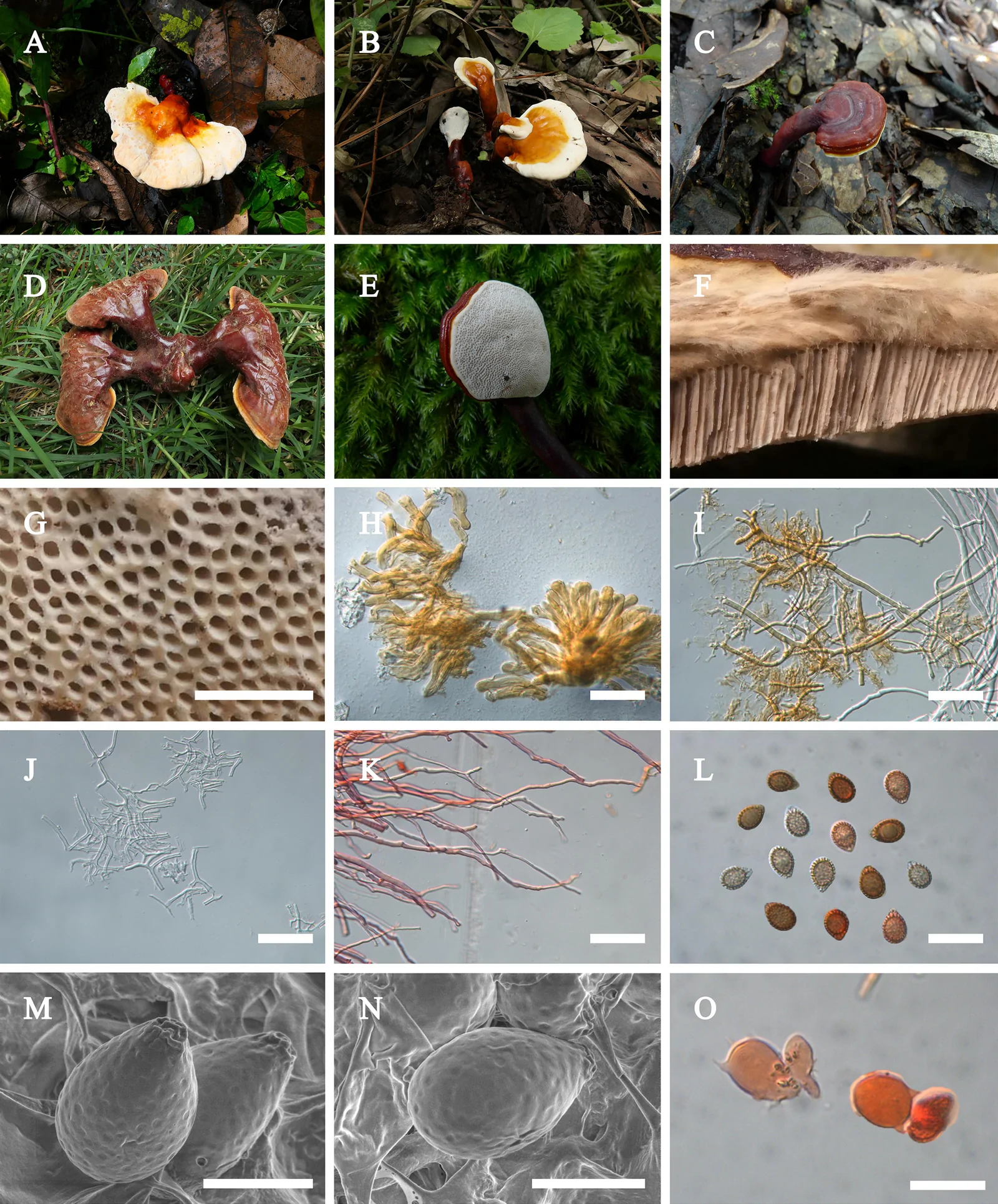
Macroscopic and microscopic features of *Ganoderma leucocontextum* (MHKMU TLP-2586). **A–E**. Basidiomata; **F**. Cut side of pileus; **G**. Pore surface; **H**. Section of pileipellis; **I**. Skeletal hyphae from context; **J**. Generative hyphae from tubes; **K**. Binding hyphae from context; **L**. Basidiospores; **M**, **N**. Basidiospores (SEM); **O**. Basidia. Scale bars: 1 cm (**G**); 50 μm (**H–K**); 20 μm (**L, O**); 5 μm (**M, N**).

##### Chinese name.

白肉灵芝.

Based on the protologue (Li et al. 2014; Ye et al. 2019) and our newly collected specimens.

##### Description.

***Basidiomata*** annual, stipitate, corky to woody. ***Pileus*** up to 25 cm in diam., and 3 cm thick at the base, variable in shape, spathulate to circular. *Pileus surface* white when young, becoming orange-yellow then reddish-brown to dark brown at maturity, with strong laccate luster; surface finely concentrically sulcate; margin acute. ***Context*** up to 2.5 cm thick, cream-white, azonate, soft, lacking black melanoid lines. ***Tube*** up to 0.4 cm long, unstratified, cream-white to pale yellowish-brown, hard-corky. ***Pores*** 4–6 per mm, subcircular; dissepiments slightly thick, entire; pore surface white when fresh, turning pale brown upon injury, cream when dry. ***Stipe*** up to 19 cm long and 1 cm in diam., lateral to dorsilateral, subcylindrical, reddish-brown.

***Hyphal system*: *Generative hyphae*** 2.0–3.5 μm in diam., with clamp connections, thin-walled, rare; ***Skeletal hyphae*** 1.5–5.0 μm in diam., thick-walled to subsolid, yellowish-brown, frequently dichotomously branched, dominant; ***Binding hyphae*** 1.0–3.0 μm in diam., thick-walled to subsolid, pale brown, with narrow lumen; IKI–, CB+.

***Basidiospores***: [40/2/2] 9.5–12.5(–13.0) × 6.5–8.5(–9.0) μm, *L* = 11.48 μm, *W* = 7.76 μm, *Q* = 1.27–1.67, *Q_m_* = 1.49 ± 0.08 (excluding myxosporium); ovoid, with indistinct apiculus, yellowish brown, IKI–; CB+, double-walled. Under light microscopy, the exospore smooth, and the endospore wall bearing moderately rough punctate ornamentation; under scanning electron microscopy exospore, ornamented with rounded to irregular pits. ***Basidia*** 14–24 × 7–12 µm, broadly clavate to fusiform, hyaline, with 2–4 sterigmata.

##### Basidiospores of *Ganoderma weixiense*

**(holotype)**. [20/1/1] 9.5–12.5 × 6.5–8.5 μm, *L* = 10.97 μm, *W* = 7.55 μm, *Q* = 1.29–1.63; *Q_m_* = 1.45 ± 0.08 (excluding myxosporium); broadly ellipsoid, apically truncate, brown, IKI–; CB+, double-walled. under light microscopy, the exospore hyaline and smooth, and the endospore with finely verrucose ornamentation.

##### Specimens examined.

**China** • Guizhou Province: Liupanshui City (六盘水市), Yusha Forest Park (玉舍森林公园), 26°27'22"N, 104°46'47"E, elev. 2,030 m, 21 August 2018, on dead wood of mixed coniferous and broad-leaved forests, *S. D. Yang* (MHKMU YSD-485); • Yunnan Province: Nanhua County (南华县), Chahe Sanjia Village (岔河三家村), 25°18'48"N, 101°16'15"E, elev. 2,080 m, 2 August 2020, on dead wood of mixed coniferous and broad-leaved forest dominated by *Pinus yunnanensis* and *Rosaceae* plants, with few *Fagaceae* plants, *W. H. Zhang* (MHKMU ZWH-321); • Shizong County (师宗县), Junzi Mountain Scenic Area (菌子山景区), 24°39'27"N, 104°10'17"E, elev. 2,200 m, 24 August 2018, on dead wood of the evergreen broad-leaved forests of the *Fagaceae*, *Ericaceae*, *Juglandaceae*. *H. Y. Huang* (MHKMU HHY-167); • same location and date, *L.P. Tang* (MHKMU TLP-2586, TLP-2701); *M. Mu* (MHKMU MM-215, MM-235); *W. H. Zhang* (MHKMU ZWH-089); Shangri-La City (香格里拉市), Yi Jia Village (彝家村), 27°23'3"N, 104°10'17"E, elev. 3,210 m, 25 August 2020, on dead wood of the mixed coniferous and broad-leaved forest dominated by *Pinus armandii*, *Quercus fabri*, and *Rhododendron decorum*, *Y. J. Pu* (MHKMU PYJ-037, PYJ-386). Xizang Autonomous Region: Linzhi City (林芝市), 29°26'3"N, 95°56'35"E, elev. 3,000 m, 15 May 2024, on dead wood, *H. C. Lei* (MHKMU LHC-133).

The holotype *of Ganoderma leucocontextum*: Xizang Autonomous Region: Bomi County (波密县), 24 August 2011, on dead wood of *Cyclobalanopsis glauca* H. P. Hu and *T. Tsering* (GDGM 40200, Holotype).

The specimens of *Ganoderma weixiense*: Yunnan Province: Weixi Lisu Autonomous County (维西傈僳族自治县), elev. 2,800 m, 24 July 2015, on dead wood in an evergreen coniferous forest, *Lei Ye* (HKAS 100649, Holotype; HKAS 100650).

##### Known distribution.

Asia—China, restricted to Southwestern provinces: Guizhou, Sichuan, Yunnan, and Xizang; at elevations of 2,000–3,300 m.

##### Notes.

*Ganoderma leucocontextum* was first described from Xizang (Li et al. 2014), and *G. weixiense* was later described from Yunnan Province (Ye et al. 2019). In our phylogenetic analysis, the *G. weixiense* sequence nested within the *G. leucocontextum* clade. Re-examination of the ITS sequences from both holotypes shows that the few observed differences are confined to the conserved 5'-end region, while all other sequences identified as *G. leucocontextum* are highly consistent. Multi-locus comparisons (nrLSU and *TEF1-α*) of four newly collected *G. leucocontextum* specimens reveal minimal nucleotide variation (< 2 bp). Morphologically, the two species are nearly identical, with basidiospore size the only reported diagnostic character. Ye et al. (2019) described *G. weixiense* basidiospore size as 6–8 × 3–4 µm (as originally published), distinctly smaller than that of *G. leucocontextum* (9.5–12.5 × 7–9 μm). However, re-measurement of the *G. weixiense* holotype yielded spore dimensions of 8.5–12.5 × 6.5–9 µm, which fall within the range of *G. leucocontextum*. The substantial discrepancy between our re-measurements of the holotype (8.5–12.5 × 6.5–9 µm) and its original description (6–8 × 3–4 µm; Ye et al. 2019) likely results from the original authors having measured immature spores. Our measurements, based on mature and fully developed basidiospores, overlap completely with those of *G. leucocontextum*, supporting their conspecificity. Based on molecular, geographic, and morphological evidence, we propose that *G. weixiense* be synonymized with *G. leucocontextum*.

Based on our field observations, *G. leucocontextum* is characterized by its solitary basidiomata (pileus up to 25 cm in diam.), with a long, distinctly lateral to eccentric stipe; a light and soft context (corky but notably lightweight compared to other laccate species); creamy-white context and hymenial surfaces; cream-white pore surfaces featuring circular to ellipsoid pores with thick, well-developed dissepiments (0.05–0.15 mm thick); an association with *Fagaceae*; and a high-altitude distribution in southwestern China.

#### Ganoderma lucidum

Taxon classificationFungiPolyporalesPolyporaceae

(Curtis) P. Karst., mycol., Toulouse 3 (No. 9): 17 (1881)

1188AFE4-0AAB-58CF-8422-47CD96632A0D

Fig. 4 A–L

##### Chinese name.

亮盖灵芝.

**Figure 4.**
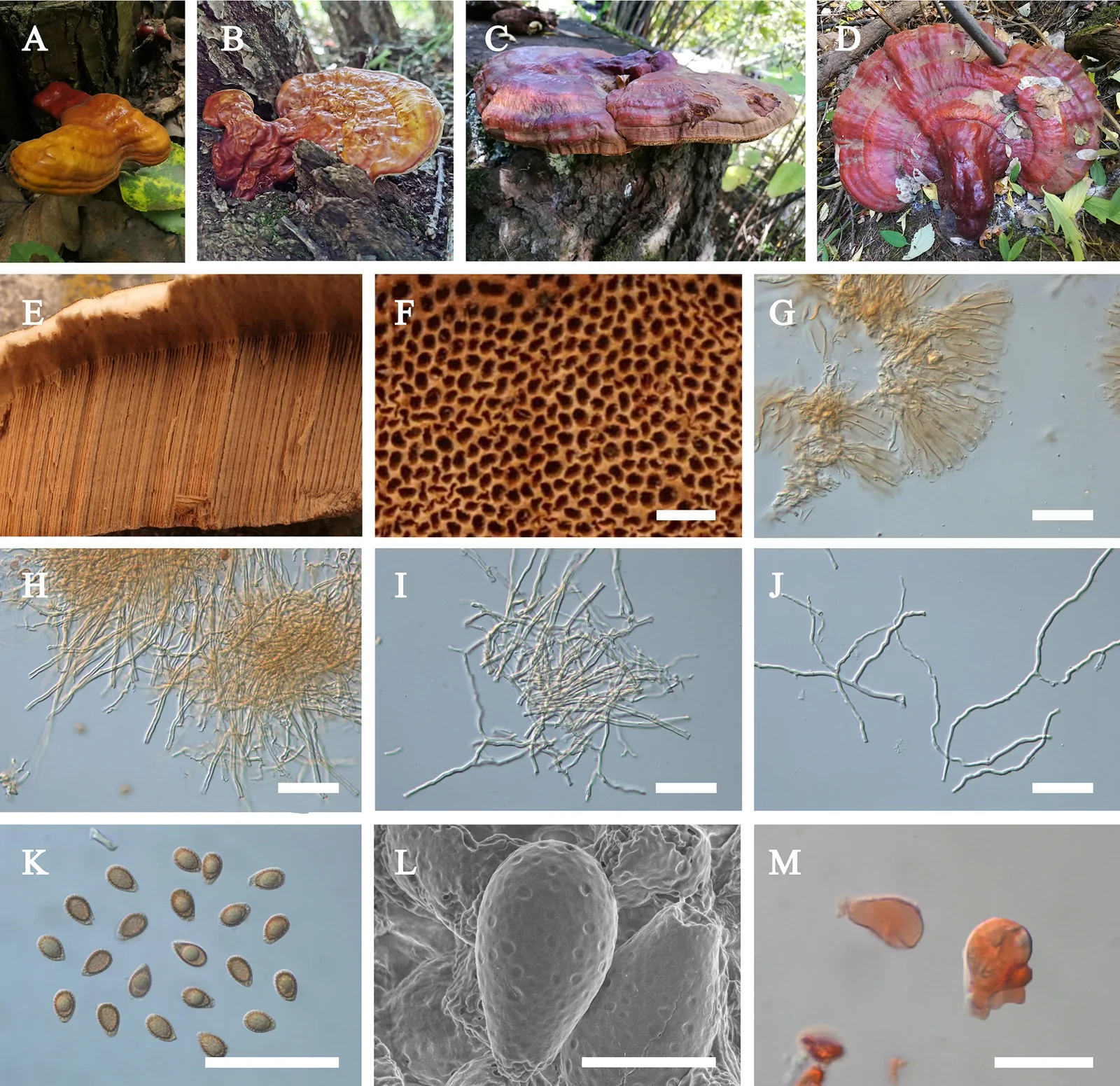
Macroscopic and microscopic features of *Ganoderma lucidum* (MHKMU MM-343, CFSZ 18739, and CFSZ 21781). **A**. Basidiomata of MHKMU MM-343 (on dead wood of mixed coniferous and broad-leaved forests dominated by birch and pine); **B**. Basidiomata of CFSZ 21781 (on rotten stumps of *Quercus mongolica*); **C**, **D**. Basidiomata of CFSZ 18739 (on rotten stumps of *Larix gmelinii*); **E**. Cut side of pileus; **F**. Pore surface; **G**. Sections of pileipellis; **H**. Skeletal hyphae from context; **I**. Generative hyphae from tubes; **J**. Binding hyphae from context; **K**. Basidiospores; **L**. Basidiospores (SEM); **M**. Basidia. Scale bars: 1 cm (**F**); 50 μm (**G–J**); 20 μm (**K, M**); 5 μm (**L**).

Based on the protologue (Xing 2019) and this study.

##### Description.

***Basidiomata*** annual, stipitate. ***Pileus*** up to 15 × 8 cm and 3 cm thick at the base, semicircular to circular, solitary. *Pileus surface* yellowish-brown to reddish-brown, with strong laccate luster, concentrically zonate, margin obtuse. ***Context*** up to 1.8 cm thick, corky to spongy, layered, cream-white or light brown above, light brown to brown near the tubes, lacking black melanoid lines. ***Tube*** up to 1.2 cm long, concolorous with context, unstratified, corky. ***Pores*** 5–6 per mm, angular to subcircular, entire, thick-walled; pore surface white when fresh, turning brown upon injury, sometimes pale yellow when dry. ***Stipe*** up to 5 cm long and 3 cm in diam., flattened to subcylindrical, lateral to eccentric, reddish-brown to purplish-black.

***Hyphal system*: *Generative hyphae*** 2.5–4 μm in diam., with clamp connections, thin-walled, hyaline, rare; ***Skeletal hyphae*** 2–10 μm in diam., thick-walled to subsolid, yellowish-brown, frequently dichotomously branched; ***Binding hyphae*** 1–3 μm in diam., thick-walled, pale brown; IKI–, CB+.

***Basidiospores***: [40/2/2] (9.0–)9.5–11.5(–12.0) × (6.0–)6.5–7.5(–8.0) μm, *L* = 10.55 μm, *W* = 7.11 μm, *Q* = 1.35–1.70, *Q_m_* = 1.60 ± 0.09 (excluding myxosporium); ellipsoid, apex truncate at maturity, yellowish brown, IKI–; CB+, double-walled. Under light microscopy, the exospore smooth, and the endospore wall bearing rough punctate ornamentation; under scanning electron microscopy exospore, ornamented with rounded to irregular pits. ***Basidia*** 10–21 × 8–12 µm, clavate, hyaline.

##### Specimens examined.

**China** • Inner Mongolia Autonomous Region: Chifeng City (赤峰市), Saihanwula National Nature Reserve (赛罕乌拉国家级自然保护区), 43°59'34"N, 118°18'36"E, elev. 900 m, 31 August 2017, on rotten stumps of *Larix gmelinii*, *T. Z. Liu* and *Wulantuya* (CFSZ 18739); • Huanggangliang Forest Farm (黄岗梁林场), 30 July 2019, on rotten stumps of *Quercus mongolica*, *Y. H. Tan* (CFSZ 21781). The Northeast Asia Botanical Garden of Changbai Mountain (长白山东北亚植物园), 42°22'54"N, 128°1'27"E, elev. 740 m, 8 August 2019, on dead wood of mixed coniferous and broad-leaved forest dominated by birch and pine, *M. Mu* (MHKMU MM-343).

##### Known distribution.

Asia—northern China (Inner Mongolia, Jilin), based on this study and previous reports. Europe—confirmed from several European countries and adjacent temperate regions (Moncalvo and Ryvarden 1997; Xing 2019).

##### Notes.

*Ganoderma lucidum* is characterized by a solitary, strongly lacquered pileus (up to 15 cm in diam.) with a yellowish (not white) immature growing margin, a pale reddish brown pore surface and context, a short and thick stipe (5 × 3 cm), and a distribution restricted to temperate regions.

As the type species of the genus, *G. lucidum* was originally described from London, UK. Historically, the name has been widely used for morphologically similar laccate specimens across multiple provinces in China (Xing 2019; Cui et al. 2023; this study). Recent revisions, however, have demonstrated that most Chinese records represent distinct species rather than the European *G. lucidum*. For instance, in Shanxi Province, specimens previously identified as *G. lucidum* were reassigned to *G. sichuanense*, *G. tsugae* Murrill, and *G. shanxiense* (Liu et al. 2024). Similarly, our phylogenetic analysis revealed that specimens from southwestern China and Thailand, long misidentified as *G. lucidum*, belong to a new species described herein as *G. sinolucidum* (see relevant section for details). The true *G. lucidum* is thus confirmed only from Europe and adjacent regions (e.g., northern China, Inner Mongolia, and Jilin); its occurrence in other temperate regions awaits further verification.

Ecologically, *G. lucidum* in Europe typically grows on dead broad-leaved trees, mainly *Fagaceae* (*Quercus* L., *Castanea* Mill., *Fagus* L.), or in mixed forests. In China, two specimens from Inner Mongolia examined in this study were collected from decayed wood of larch (*Larix gmelinii*) and Mongolian oak (*Quercus mongolica*), respectively, suggesting that the substrate preference of *G. lucidum* may be broader than traditionally recognized, at least in northern Asian populations.

#### Ganoderma oregonense

Taxon classificationFungiPolyporalesPolyporaceae

Murrill, N. Amer. Fl (New York) 9(2): 119 (1908) syn. nov.

B6E5CEED-0DE7-51F1-AEAE-E72BC4BCD842

Fig. 5 A–C

##### Chinese name.

俄勒冈灵芝.

**Figure 5.**
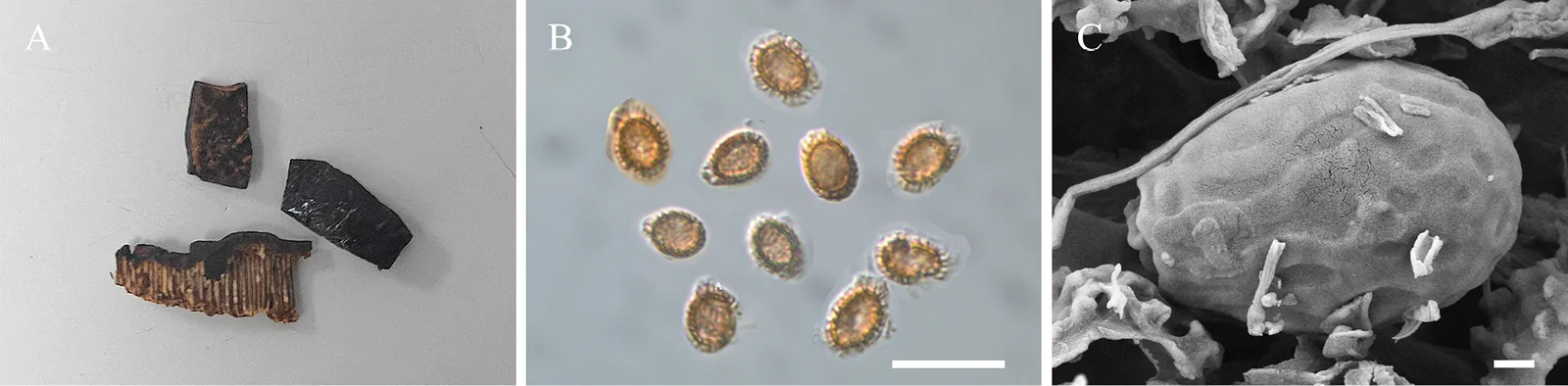
Macroscopic and microscopic features of *Ganoderma tibetanum* (HMAS 41261, holotype). **A**. Basidiomata; **B**. Basidiospores; **C**. Basidiospores (SEM). Scale bars: 20 μm (**B**); 1 μm (**C**).

##### Synonym.

*Ganoderma tibetanum* J.D. Zhao & X.Q. Zhang

Because the holotype of *Ganoderma tibetanum* (HMAS 41261) is malformed with an underdeveloped cuticle, a complete morphological description cannot be obtained directly from this specimen. Given the limited morphological data available from the Chinese specimen, the following description is primarily synthesized from the North American protologue (Torres-Torres et al. 2015), supplemented by our spore measurements from the holotype:

##### Description.

***Basidiomata*** annual, mainly sessile to substipitate. ***Pileus*** 7–23 × 10–30 cm and 1.2 cm thick at the base, rounded-flabelliform, solitary or rarely imbricate. *Pileus surface* reddish-brown, purplish-brown, dark brown to nearly black, margin lighter than the base to concolorous; dull to semiglossy, cracked after drying, azonate to slightly zonate; ***Context*** 2.5–10 mm thick, soft-corky, layered, orange-white or cream-orange-pink above, light brown to raw-sienna near the tubes. ***Tube*** up to 0.3–0.6 cm long, almost concolorous with the lower part of the context, fragile. ***Pores*** 3–4 per mm; pore surface yellowish-white to orange-brown or raw-sienna. ***Stipe*** 4–13 cm long and 4–4.5 cm in diam., cylindrical; reddish-black, darker than pileus.

***Hyphal system*: *Generative hyphae*** 2.5–5.5 μm in diam., with clamped connections; ***Skeletal hyphae*** 2–10 μm in diam., thick-walled, non-branched to arboriform; ***Binding hyphae*** 3–8 µm in diam., thick-walled; IKI–, CB+.

##### Basidiospores of *Ganoderma tibetanum*

**(holotype)**. [20/1/1] 11.0–14.0(–14.5) × (7.0–)7.5–9.5 μm, *L* = 13.15 μm, *W* = 8.65 μm, *Q* = 1.39–1.60, *Q_m_* = 1.45 ± 0.08 (excluding myxosporium); ellipsoid to oblong, apex subacute, yellowish brown, inamyloid (IKI–); cyanophilous (CB+), double-walled. Under light microscopy, the exospore smooth, and the endospore walls bearing interwall pillars; under scanning electron microscopy, the surface of the exospore wall has slight irregular depressions. ***Basidia*** 24–40 × 7–9 µm, clavate, hyaline.

##### Specimens examined.

**China** • Xizang Autonomous Region: Lixin Township (立新乡), elev. 2,550 m, 18 July 1975, on dead wood of *Quercus semicarpifolia*, He 56 (HMAS 41261, Holotype); • Hainan Province: Bawangling (坝王岭), (HMAS 57943).

##### Known distribution.

North America—Canada, USA, Cuba (Murrill 1908; Hong and Jung 2004; Milay et al. 2023). Asia—China (Xizang Autonomous Region), confirmed by sequencing the holotype of *Ganoderma tibetanum* (this study). This is the first verified Asian record.

##### Notes.

*Ganoderma tibetanum* was described from the Xizang Autonomous Region, China (Zhao et al. 1983). Since its publication, this species has been documented in only two studies (Zhao and Zhang 2000; Wu and Dai 2005), both of which recorded it from Hainan Province, in addition to the type locality in Xizang. It has been considered a taxonomically doubtful species within the genus *Ganoderma*, with its status remaining unresolved.

We examined both the holotype (Xizang) and the Hainan specimen referred to this species. The Hainan specimen was heavily damaged by insects; no basidiospores were observed under SEM, and no other useful morphological features were present. Further, we were unable to obtain DNA sequences from this specimen. The identity of the Hainan collection remains unclear. The holotype from Xizang is malformed, with an irregular pileus and underdeveloped cuticle and spores, rendering reliable morphological identification impossible. Moreover, its DNA is highly degraded, and conventional Sanger sequencing repeatedly failed.

In this study, we successfully obtained the ITS sequence from the holotype using next-generation sequencing (GenBank accession PZ537036). The obtained sequence was of good quality, clearly alignable, and phylogenetically informative. Phylogenetic analysis places this sequence within the *G. oregonense* clade with moderate to good support (BS = 75%, PP = 0.95) (Fig. 1). Although this bootstrap value falls below the conventional 95% threshold for “strong” support, it exceeds the commonly accepted 70% cutoff for “moderate” support (Hillis and Bull 1993). Critically, the basidiospores of the holotype–the only morphologically informative feature available–measure 11.0–14.0 × 7.5–9.5 µm with ellipsoid to oblong shape and a subacute apex (Fig. 5B, C), falling entirely within the range reported for *G. oregonense* (Torres-Torres et al. 2015). In addition, the known occurrence of *G. oregonense* in North America is disjunct but ecologically analogous to the habitat in Xizang. While the absence of complete morphological data is an acknowledged limitation, the ITS phylogeny, basidiospore morphology, and habitat all support the conspecificity of *G. tibetanum* with *G. oregonense*. Obtaining the ITS sequence not only resolved the identity of this long-standing doubtful species but also confirmed the occurrence of *G. oregonense* in China. Accordingly, we treat *G. tibetanum* as a synonym of *G. oregonense*. As a result, *G. oregonense* is reported here for the first time from China, representing one of the few documented disjunct distributions between Asia and North America in the genus *Ganoderma*. To date, only two species, *G. applanatum* (Pers.) Pat. and *G. tsugae* Murrill, have been documented with transcontinental distributions across Asia and North America (Wu and Dai 2005; Xing 2019); this study adds *G. oregonense* as the third such species.

#### Ganoderma roseogelatum

Taxon classificationFungiPolyporalesPolyporaceae

L.P. Tang, H.C. Lei, J. Li & Z.H. Wu sp. nov.

8603C1E2-27FC-5745-A0E8-A220678C3F7C

861461

Fig. 6 A–R

##### Chinese name.

粉胶灵芝.

**Figure 6.**
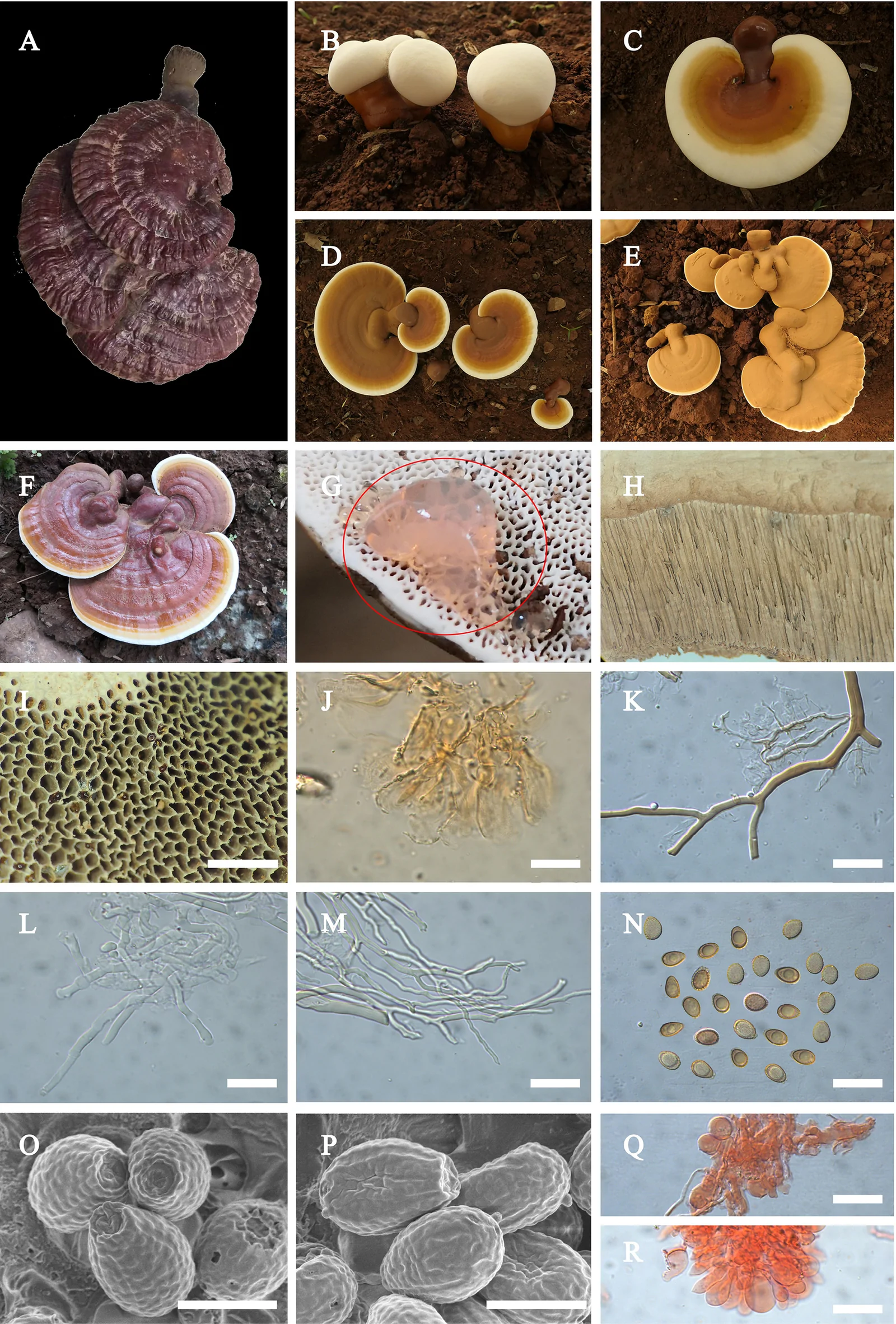
Macroscopic and microscopic features of *Ganoderma roseogelatum* (MHKMU LHC-184, holotype). **A–G**. Basidiomata; **H**. Cut side of pileus; **I**. Pore surface; **J**. Section of pileipellis; **K**. Skeletal hyphae from context; **L**. Generative hyphae from tubes; **M**. Binding hyphae from context; **N**. Basidiospores; **O**, **P**. Basidiospores (SEM); **Q**, **R**. Basidia and basidioles. Scale bars: 1 cm (**I**); 20 μm (**J–N, Q, R**); 5 μm (**O, P**).

##### Etymology.

*Roseogelatum* (Latin) refers to the fact that fresh basidiomata of this species slowly secrete a pinkish (roseus) gelatinous (gelatus) substance upon injury, a distinctive feature not yet observed in any other known species of *Ganoderma*.

##### Diagnosis.

Distinguished from other *Ganoderma* species by the following combination of characters: basidiomata with a lacquer-like shine and a short, often lateral stipe; pileus usually multiple and complex in shape, nearly flat-spherical to hemispherical when young with a white growing margin; pore surface white; pores predominantly irregular, with thin and poorly developed dissepiments; basidiospores distinctly truncated, with a tuberculate exospore wall under SEM, the tubercles regularly arranged longitudinally; context distinctly fragrant, without distinct bitterness.

##### Holotype.

**China** • Yunnan Province, Xinping County (新平县), Gasa Town (戛洒镇), 24°03'34"N, 101°34'29"E, elev. 1,500 m, 28 July 2023, *H.C. Lei* (MHKMU LHC-184, holotype).

##### Description.

***Basidiomata*** annual, stipitate, hard and corky. ***Pileus*** up to 20 × 10 cm, 2.3–4.0 cm thick at the base, often concrescent, forming compound irregular pilei; shape nearly flat-spherical to hemispherical when young, becoming semicircular, sub-circular, or nearly reniform at maturity. ***Pileus surface*** white (1A1) when young, maturing to grape-brown (9D5) or brownish red (10E5), drying dark brown (11F7); surface covered with a thin hard crust, weakly to strongly laccate, with concentric furrows and radial wrinkles; margin entire, acute to obtuse, slightly wavy. ***Context*** 1.5–3.5 cm thick, heterogeneous: upper layer light brown (3B2), dark brown (5E5) above the tubes, showing concentric growth zones at the base, lacking black melanoid lines, corky. ***Tubes*** 0.6–1 cm long, dark brown (5E5), corky, unstratified. ***Pores*** 3–4 per mm (0.20–0.45 mm in diam.), predominantly irregular, dissepiments poorly developed, up to 0.1 mm thick; pore surface off-white (1A1) when fresh, turning sorrel (9E4) upon bruising and exuding a pale pink (6A2) gelatinous substance in all examined fresh specimens, becoming dark brown (6E5) when dried. ***Stipe*** short and thick, 2–4 cm long and 2–3 cm in diam., nearly cylindrical, lateral; light orange-yellow (5A5) when young, becoming brownish red (10E5) at maturity, strongly laccate; texture soft corky. ***Odor*** fragrant, taste not distinctly bitter.

***Hyphal system trimitic*. *Generative hyphae*** 1–7 μm in diam., colorless, hyaline, thin-walled, abundant, with clamp connections. ***Skeletal hyphae*** 2–10 μm in diam., chartreuse (30A2) to orange-brown (6C4), thick-walled with a narrow lumen to sub-solid, occasionally branched, predominant. ***Binding hyphae*** 1–4 μm in diam., colorless to chartreuse (30A2), sub-thick-walled to solid, frequently branched, interwoven. All hyphae IKI–, CB+; tissues darkening in KOH.

***Pileipellis***: a crustohymenidermis. Cells 23–52 × 7–19 μm, clavate to broadly clavate, yellowish to yellowish brown, thick-walled to sub-solid, occasionally with granulations at the apex, arranged in a regular palisade; apical part negative or slightly amyloid.

***Basidiospores***: [40/2/2] (8.0–)8.5–11.0(–12.5) × (6.0–)6.5–8.5(–9.5) µm, *L* = 9.84 μm, *W* = 7.33 μm, *Q* = 1.17–1.50 (excluding myxosporium), *Q_m_* = 1.35 ± 0.10; almond-shaped, broadly ellipsoid to ovoid, with a conspicuously truncated apex; yellowish to yellowish brown (6D7), inamyloid (IKI–), cyanophilous (CB+); double-walled with distinctly thick walls, exospore wall smooth; under light microscope, endospore wall with dense spinules; under scanning electron microscopy, exospore tuberculate, ornamentation composed of numerous, typically isolated tubercles arranged longitudinally into low ridges. ***Basidia***: 13–15 × 7–11 μm, broadly clavate to fusiform, colorless, thin-walled, 2- or 4-sterigmate; basidioles 9–26 × 5–13 μm, fusiform, clavate to broadly clavate, colorless, thin-walled.

##### Additional specimens examined

**(paratypes). China** • Yunnan Province, Lufeng County (禄丰县), Caiyun Town (彩云镇), 25°02'29"N, 101°52'49"E, elev. 1,400 m, 28 July 2024, cultivated on wood segments, *H. C. Lei* (MHKMU LHC-130, LHC-137, LHC-185, paratypes).

##### Known distribution.

Asia—China, confirmed with morphological vouchers only from Yunnan Province. Additional sequences from Sichuan Province (China) and South Korea also cluster with clade (see Fig. 1), suggesting a potentially wider distribution, but these records require field verification with corresponding specimens.

##### Notes.

*Ganoderma roseogelatum* is sold in the medicinal materials market of Gasa Town, Yunnan Province, China. Local traditional healers of the Bai and Yi ethnic groups regard this fungus as a medicinal mushroom (“Lingzhi”) and use it to treat insomnia, hangovers, and various cancers. This species is also represented by GenBank accessions from Sichuan, China, and South Korea (see Table 1). However, as these sequences lack corresponding voucher specimens for morphological verification, the confirmed distribution of *G. roseogelatum* currently remains restricted to Yunnan Province until further collections are obtained. Additional field surveys are needed to verify its natural occurrence in these regions and to clarify its substrate preference.

Morphologically, *G. roseogelatum* is readily distinguished from all similar taxa by its typically multiple and intricately fused pilei, a feature not observed in any laccate *Ganoderma* species in China. Furthermore, the stipe of *G. roseogelatum* is extremely short and lateral, often inconspicuous, and mostly lateral, giving the basidiomata a nearly sessile appearance. Young specimens exhibit a white growing margin and an off-white pore surface, with irregular, angular to lacerate pores and poorly developed dissepiments. A unique and diagnostically valuable trait is the exudation of a pinkish gelatinous substance upon injury, which was consistently observed in all examined fresh specimens, and has not been observed in other *Ganoderma* species.

Among Chinese taxa, *Ganoderma leucocontextum*—also distributed in southwestern China but at higher elevation (2,000–3,300 m)—is readily distinguished by its solitary basidiomata with a long, well-developed stipe, a strongly lacquered pileus surface, circular and entire pores with well-developed dissepiments, and a notably light and soft texture. Ecologically, it grows on dead wood of broad-leaved trees (mainly *Fagaceae*), whereas the substrate preference of *G. roseogelatum* remains unclear.

*Ganoderma lucidum*, originally described from Europe, is mainly found in temperate regions of northern China (e.g., Inner Mongolia and Jilin) (Ryvarden and Gilbertson 1993; Ye et al. 2019; Sun et al. 2022; this study). It produces a solitary, strongly lacquered pileus (up to 15 cm in diam.) with a short, lateral stipe, a yellowish (not white) growing margin when immature, a pale reddish brown pore surface and heavy context, and basidiospores that are slightly narrower and longer (10.5–12.5 × 6.5–7.5 μm) than those of *G. roseogelatum* (Ryvarden and Gilbertson 1993; Ye et al. 2019; Sun et al. 2022; this study). Furthermore, its pores are red-brown and polygonal, with slightly thick but well-developed dissepiments (Fig. 4).

*Ganoderma roseogelatum* is morphologically reminiscent of *Ganoderma tropicum* (Jungh.) Bres., a species originally from Java and distributed in tropical regions, with which it shares a similarly short, lateral stipe, a reddish-brown pileus up to 20 cm in diameter, and occurrence in the same local markets of Xinping County, Yunnan. However, *G. tropicum* is readily distinguished by its pore surface color pale yellow to light yellow, turning brown to dark brown when touched; a context that is light yellow to yellow near the crust and grayish yellow to brown near the tubes; and much smaller pores (0.065–0.120 mm in diameter) (Luangharn et al. 2019).

Phylogenetically, *G. roseogelatum* is sister to *G. resinaceum* from Europe, but the latter is perennial and sessile, with a strongly lacquered surface and regular, circular pores with well-developed dissepiments (Steyaert 1972).

#### Ganoderma sinolucidum

Taxon classificationFungiPolyporalesPolyporaceae

L.P. Tang, H.C. Lei, J. Li & Z.H. Wu sp. nov.

EEC4B9BB-144A-5982-8366-8E28669AEAB9

861462

Fig. 7 A–O

##### Chinese name.

中华亮盖灵芝.

**Figure 7.**
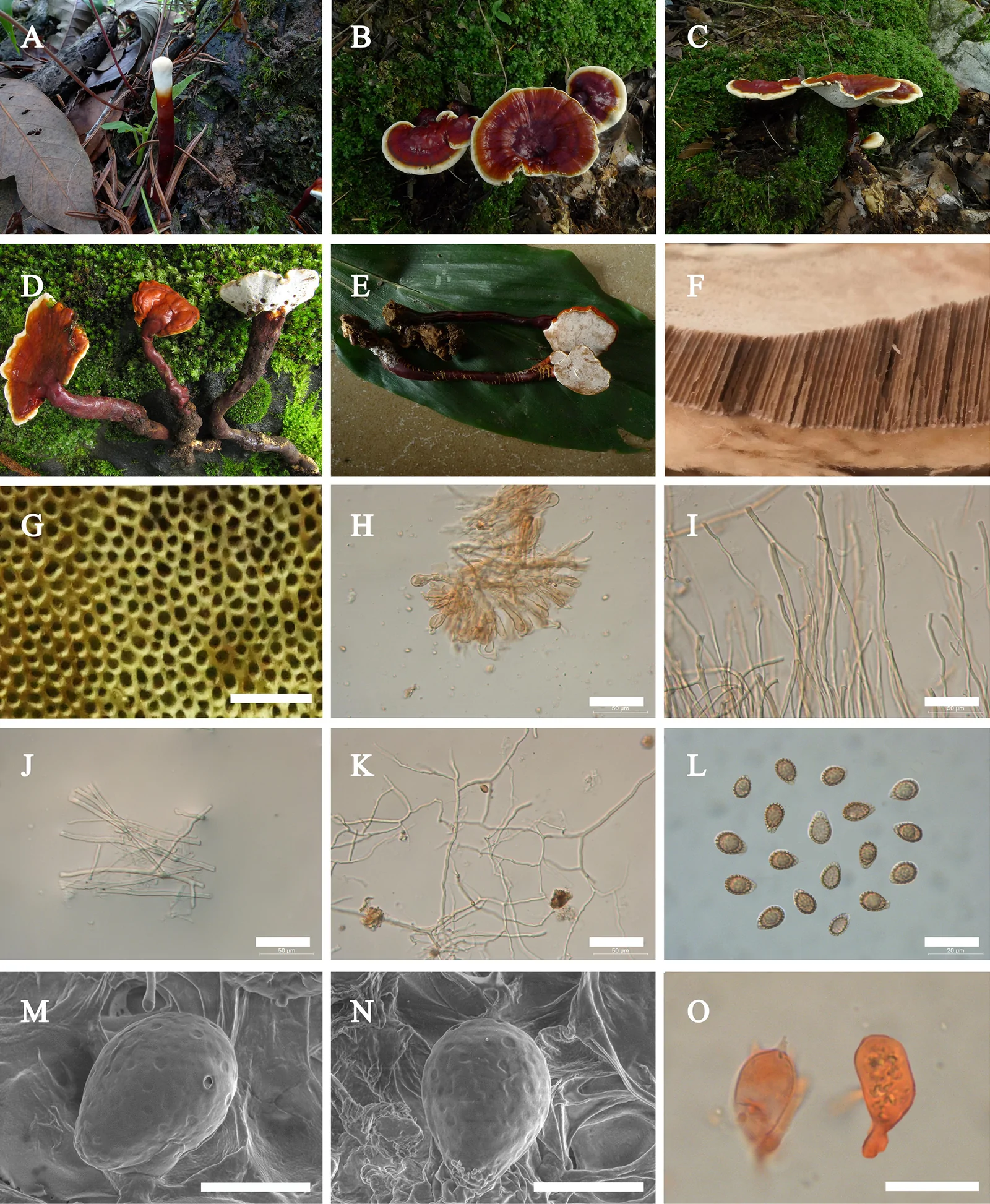
Macroscopic and microscopic features of *Ganoderma sinolucidum* (MHKMU MJ-06, holotype). **A–E**. Basidiomata; **F**. Cut side of pileus; **G**. Pore surface; **H**. Section of pileipellis; **I**. Skeletal hyphae from context; **J**. Generative hyphae from tubes; **K**. Binding hyphae from context; **L**. Basidiospores; **M**, **N**. Basidiospores (SEM); **O**. Basidia. Scale bars: 1 cm (**G**); 50 μm (**H–K**); 20 μm (**L, O**); 5 μm (**M, N**).

##### Etymology.

*Sinolucidum* refers to a species morphologically similar to *G. lucidum* and distributed in China.

##### Diagnosis.

Distinguished from other species of *Ganoderma* by the following combination of characters: basidiomata usually solitary, with a lacquer-like shine and a mostly lateral, elongated, slender stipe that is initially white at the apex; pore surface and context persistently milky white even at maturity; pores predominantly subcircular and entire, with well-developed dissepiments; basidiospores relatively rounder, 7.5–13.5 × 6–9 μm, slightly or non-truncate; occurring exclusively on broad-leaved trees; distributed in tropical to subtropical regions of Asia, particularly in China and Thailand.

##### Holotype.

**China** • Yunnan Province: Qujing City (曲靖市), Haizhai Forestry (海寨林场), 25°15'1"N, 103°59'19"E, elev. 2,130 m, 2 October 2021, on dead wood of evergreen broad-leaved forest, *J. Ma* (MHKMU MJ-06).

##### Description.

***Basidiomata*** annual, stipitate, corky. ***Pileus*** 2.0–7.0 × 3.5–4.5 cm, 3.0–4.0 cm thick at base, solitary, subcircular to flabelliform or reniform at maturity. ***Pileus surface*** white when young, maturing to grape-brown (9D5) or brownish red (10D8); surface smooth, covered by a thin, hard crust with a lacquer-like luster, concentrically zonate or azonate; margin concolorous to creamy (1A2), acute to obtuse, slightly wavy. ***Context*** 0.3 cm thick, homogeneous, milky white (7A1), lacking black melanoid lines, corky. ***Tubes*** 0.2–1.0 cm long, lengthening with age, brownish (9D5), corky, unstratified. ***Pores*** 3–4 per mm (0.14–0.32 mm in diam.), mostly circular, subcircular, a few angular, entire; dissepiments well developed, 0.03–0.13 mm thick; pore surface milky white (7A1) to light brown (6B2) when fresh, becoming light gray (8B1) when dried. ***Stipe*** mostly long and slender, 5–16 × 0.2–2.5 cm, cylindrical; mostly lateral, rarely central or eccentric; initially slightly swollen at the apex, becoming nearly equal at maturity; reddish brown (9E1) to purple-brown (10E5); strongly laccate; texture soft corky.

***Hyphal system trimitic*. *Generative hyphae*** 3–4 μm in diam., colorless, hyaline, thin-walled, abundant, with clamp connections. ***Skeletal hyphae*** 4–7 μm in diam., brownish yellow (5B4), thick-walled to subsolid, often dichotomously branched, predominant. ***Binding hyphae*** 1–2 μm in diam., colorless, thick-walled with a narrow lumen to nearly solid, frequently tortuous and branched. All the hyphae IKI–, CB+; tissues darkening in KOH.

***Pileipellis***: a crustohymenidermis, cells 25–50 × 8–13 μm, regular palisade, apex slightly thicker than base, yellowish-brown (5B6), thick-walled to subsolid, vertically and compactly arranged; in mature basidiomata, cuticle cells typically show faint to strong amyloid reactions.

***Basidiospores***: [40/2/2] (9.0–)9.5–13.5(–14) × 6.0–9.0 (–9.5) μm, *L* = 11.87 μm, *W* = 7.83 μm, *Q* = 1.33–1.69 (excluding myxosporium), *Q_m_* = 1.52 ± 0.09; ellipsoid to ovoid, inconspicuously truncate or non-truncate, yellowish to yellowish brown (6D7), inamyloid (IKI–); cyanophilous (CB+), double-walled, walls slightly thick. Under light microscopy, exospore smooth, endospore bearing interwall pillars; under scanning electron microscopy, exospore ornamented with rounded to irregular pits, isolated. ***Basidia***: 10–25 × 6–13 μm, broadly fusiform to utriform, hyaline, thin-walled, 2- or 4-sterigmate.

##### Additional specimens examined.

**China** • Yunnan Province, Shizong County (师宗县), Junzi Mountain Scenic Area (菌子山风景区), 24°44'23"N, 104°9'48"E, elev. 2,280 m, 24 August 2018, on dead wood of evergreen broad-leaved forests, *J. Ma* (MHKMU MJ-0007), *J. M. Niu* (MHKMU NJM-076); • same location, elev. 1,870 m, 21 July 2020, on dead wood of *Castanea*-dominated mixed broad-leaved forests, *M. Mu* (MHKMU MM-487); • Shizong County, Niusu Village (牛速村), 24°44'32"N, 104°5'57"E, elev. 1,820 m, 21 July 2020, on dead wood of evergreen broad-leaved forests dominated by *Rhododendron* spp. and *Castanea* spp., *L. P. Tang* (MHKMU TLP-3017).

##### Known distribution.

Asia—tropical to subtropical regions. Confirmed by voucher specimens from Yunnan, China (elev. 1,800–2,300 m). Additionally, sequences from Sichuan Province, China (MG279181), and Thailand (MN396341) are available, but the localities lack examined specimens and require future confirmation.

##### Notes.

*Ganoderma sinolucidum* is characterized by relatively small, thin basidiomata with a slender stipe; a milky white pore surface and context; subcircular, entire pores with well-developed dissepiments; occurrence on broad-leaved trees; and a distribution restricted to tropical and subtropical regions of Asia. In central Yunnan, this species is locally known as “Xue Lingzhi” (血灵芝) and has been cultivated on a small scale.

Historically, *G. sinolucidum* has been widely misidentified in Asia as the European *G. lucidum* (Xing et al. 2018; Luangharn et al. 2019; Sun et al. 2022). The two species are sister clades and share a similarly colored pileus, but *G. lucidum* (see Fig. 4) has a yellowish growing margin and differs in host association (conifers vs. broad-leaved trees) and distribution (temperate vs. tropical-subtropical).

*Ganoderma sinolucidum* may also be confused with *G. leucocontextum* and *G. tsugae* due to their superficially similar pileus coloration. Both of these species have larger basidiomata: *G. leucocontextum* differs in its larger pileus (up to 20 cm in diam.) and a short, thick stipe, sometimes almost sessile (Li et al. 2014). *Ganoderma tsugae* is restricted to conifers, has sessile to laterally stipitate basidiomata (pileus up to 30 cm in diam.), longer basidiospores (13–15 × 7.5–8.5 µm), and a pileus margin that often splits into petal-like layers (Gilbertson and Ryvarden 1986; Zhou et al. 2015).

A name from Asia, *G. ahmadii* Steyaert (Pakistan, on *Dalbergia sissoo*), lacks molecular data; its original description (thinner, dark pileus, smaller basidiospores 5.0–11.0 × 4.0–7.0 µm; Steyaert 1972) suggests it is distinct from *G. sinolucidum*, but phylogenetic confirmation is currently impossible. Based on the available morphological and molecular evidence, we recognize *G. sinolucidum* as a distinct species.

## Discussion

### Species delimitation and name application

Accurate species delimitation in *Ganoderma* is critical for both scientific research and practical application. The European name *G. lucidum* has been widely misapplied to Asian specimens for over 100 years. More than a dozen species have been referred to under this name, including *G. lingzhi*, *G. leucocontextum*, *G. multipileum*, *G. sichuanense*, *G. sinolucidum*, *G. shanxiense*, *G. tsugae*, and others (Cao et al. 2012; Wang 2012; Li et al. 2014; Zhou et al. 2015; Liu et al. 2024). Our phylogenetic and morphological evidence clearly demonstrates that true *G. lucidum* is a temperate species distributed in Europe and extending into adjacent temperate East Asia (e.g., Inner Mongolia and Jilin, China), rather than being strictly restricted to Europe as previously assumed (Moncalvo and Ryvarden 1997). In China, it grows on conifers (e.g., *Larix gmelinii*) and broad-leaved trees (e.g., *Quercus mongolica*). In contrast, specimens from southwestern China and Thailand previously identified as *G. lucidum* belong to a distinct species, here described as *G. sinolucidum* sp. nov. (locally known as “Xue Lingzhi” in Yunnan). This species is tropical to subtropical, occurs on broad-leaved trees (*Fagaceae*), and differs from *G. lucidum* in pileus margin color (white vs. yellowish-brown) and basidiospore shape.

We further clarify the taxonomic status of *G. leucocontextum* and *G. weixiense*, both described from high-altitude southwestern China based solely on ITS sequences and with overlapping distributions. Re-measurement of the *G. weixiense* holotype revealed spore dimensions falling within the range of *G. leucocontextum*, contradicting the original description. Phylogenetic analysis places *G. weixiense* within the *G. leucocontextum* clade, and multi-locus sequences from newly collected specimens show minimal divergence. Therefore, we synonymize *G. weixiense* under *G. leucocontextum* syn. nov.

The case of *G. tibetanum* demonstrates the value of sequencing type specimens for resolving long-standing taxonomic puzzles, while also illustrating the challenges inherent in working with aged herbarium materials. Since it was described from Xizang (Zhao et al. 1983), *G. tibetanum* has been reported and has long been considered a taxonomically doubtful species. In this study, we successfully obtained ITS sequence from the malformed holotype using next-generation sequencing—an approach necessitated by the repeated failure of conventional Sanger sequencing due to highly degraded DNA. The obtained sequence was of good quality, clearly alignable, and phylogenetically informative. Phylogenetic analysis places this sequence within the clade of *G. oregonense* with moderate support. While this support value does not constitute strong statistical evidence by itself, it exceeds the commonly accepted 70% cutoff for “moderate” support and is corroborated by the concordance of basidiospore morphology with the protologue of *G. oregonense* syn. nov. The convergence of these lines of evidence—though not definitive—provides the best available resolution for this problematic taxon. These findings exemplify how molecular re-examination of type specimens can uncover hidden biogeographic connections and resolve species boundaries that morphology alone cannot.

### Taxonomic value of morphological traits

#### Early developmental traits

The color of the growing margin in young basidiomata provides reliable diagnostic information that is often overlooked. In our study, *G. roseogelatum*, *G. leucocontextum*, and *G. sinolucidum* all exhibit a white growing margin, whereas *G. lucidum* has a yellowish-brown margin. Additionally, *G. roseogelatum* exudes a distinctive pinkish gelatinous substance from injured pores at the young stage, a rare trait in *Ganoderma*. We recommend that field collections document these ephemeral ontogenetic features.

#### Pore characteristics

Pore morphology differs markedly among the studied species and has high taxonomic value. Table 2 summarizes the key pore traits for quick comparison.

**Table 2.** A comparison of pore characteristics among the studied *Ganoderma* species.

**Taxa**	**Pore surface color**	**Pore shape**	**Dissepiment development**	**References**
*Ganoderma eickeri*	cream-white	triangular to quadrangular, entire	thick, well-developed	Tchotet-Tchoumi et al. 2019; this study
*G. roseogelatum*	yellowish-white	predominantly irregular	thin, poorly developed	This study
*G. leucocontextum*	cream-white	circular to ellipsoid, entire	thick, well-developed	Li et al. 2014; this study
*G. lingzhi*	Pale yellow to sulfur yellow	circular to ellipsoid, entire	slightly thick, well-developed	Cao et al. 2012; this study
*G. lucidum*	pale reddish-brown	predominantly polygonal, nearly entire	slightly thin, developed	Ye et al. 2019; Sun et al. 2022; this study
*G. sinolucidum*	milky-white	subcircular, entire	slightly thick, well-developed	This study

### Biogeographic implications

Our findings reveal contrasting distribution patterns among *Ganoderma* species. Some species exhibit narrow endemism: *G. sinolucidum* is restricted to tropical-subtropical Asia (southwestern China and Thailand), whereas *G. leucocontextum* is confined to high-altitude regions (2,000–3,300 m) of southwestern China. By contrast, true *G. lucidum* is distributed in temperate regions (Europe and adjacent East Asia); in China, it is mainly found in northeastern regions (e.g., Inner Mongolia and Jilin). In southwestern China, both *G. roseogelatum* and *G. sinolucidum* have been historically misidentified as *G. lucidum*. This study clarifies these long-standing confusions.

Two species reported here as new records for China provide significant biogeographic insights. *Ganoderma eickeri*, originally described from South Africa, is reported for the first time outside its native continent, representing a new Afro-Asian disjunction supported by molecular evidence. *Ganoderma oregonense*, originally from North America, is documented in Asia for the first time; its occurrence in China was confirmed through sequencing the holotype of the previously doubtful taxon *G. tibetanum*. Thus, *G. oregonense* becomes the third *Ganoderma* species (after *G. applanatum* and *G. tsugae*) with a confirmed transcontinental disjunction between Asia and North America (Wu and Dai 2005; Xing 2019). These findings highlight the importance of molecular re-examination for type specimens to uncover hidden biogeographic connections. However, caution is needed: many historical reports of “cosmopolitan” species are likely based on misidentifications (Tang et al. 2014; Huang et al. 2021; Yu et al. 2025, 2026). The true distribution ranges of most *Ganoderma* species remain to be fully elucidated through integrative taxonomy.

Despite the integrative approach adopted in this study, several limitations remain. Molecular data are unavailable for some morphologically similar taxa (e.g., *G. ahmadii*; Steyaert 1972), preventing their definitive phylogenetic placement. Additionally, the host ranges and ecological preferences of the newly distinguished species remain poorly understood. Future studies should prioritize multi-locus sequencing of type specimens and extensive field sampling across under-explored regions, as well as exploring the biotechnological applications of newly resolved taxa (Asad et al. 2025).

## Conclusion

This study integrates morphology (including early developmental traits and pore details) with multi-locus phylogenetics to resolve species boundaries within the *Ganoderma* genus, particularly the *G. lucidum* species complex. We describe two new species (*G. roseogelatum* sp. nov. and *G. sinolucidum* sp. nov.), report two new records for China (*G. eickeri* from Africa and *G. oregonense* from North America), and propose two synonymizations (*G. weixiense* under *G. leucocontextum* syn. nov. and *G. tibetanum* under *G. oregonense* syn. nov.). Crucially, obtaining the ITS sequence for the holotype *G. tibetanum*—a long-standing doubtful taxon—not only resolved its identity but also confirmed the presence of *G. oregonense* in China. Our results clearly identify *G. sinolucidum* as a distinct Asian species, distinct from the European *G. lucidum*. To date, 52 taxa have been recorded in China. These findings provide a robust taxonomic basis for biogeographic and pharmacological research on this economically important genus.

## Supplementary Material

XML Treatment for Ganoderma eickeri

XML Treatment for Ganoderma leucocontextum

XML Treatment for Ganoderma lucidum

XML Treatment for Ganoderma oregonense

XML Treatment for Ganoderma roseogelatum

XML Treatment for Ganoderma sinolucidum

## Additional information

### Artificial Intelligence (AI) use

The authors accept full responsibility for the content of the manuscript, including the disclosure of any use of AI.

No AI tools were used in the preparation of this manuscript.

### Author ORCIDs

H.-C. Lei https://orcid.org/0009-0009-4805-5354

J. Li https://orcid.org/0009-0007-2442-7008

T.-J. Yu https://orcid.org/0009-0003-6944-7329

Y.-X. Gao https://orcid.org/0009-0007-2276-6831

Y.-C. Dai https://orcid.org/0000-0002-6523-0320

T.-Z. Liu https://orcid.org/0000-0003-1751-2195

T. Li https://orcid.org/0009-0004-5873-8371

Y.-T. Lu https://orcid.org/0000-0002-1278-5584

L.-P. Tang https://orcid.org/0000-0002-6905-3157

### Data availability

All new sequences generated in this work have been deposited in GenBank (https://www.ncbi.nlm.nih.gov/genbank/). All other data that support the findings of this study are available in the main text and supplementary files.

## Supplementary materials

Supplementary material 1 Tree-PDF Data type pdf https://binary.pensoft.net/file/1785602This dataset is made available under the Open Database License (http://opendatacommons.org/licenses/odbl/1.0/). The Open Database License (ODbL) is a license agreement intended to allow users to freely share, modify, and use this Dataset while maintaining this same freedom for others, provided that the original source and author(s) are credited. Han-Chi Lei, Jia Li, Tai-Jie Yu, Yu-Xian Gao, Zhong-Hua Wu, Yu-Cheng Dai, Tie-Zhi Liu, Run-Ke Mao, Wei-Jian Guan, Ying Tang, Ting Li, Yuan-Tian Lu, Qing-Wei Zang, Li-Ping Tang

Supplementary material 2 Tree-treefile Data type treefile https://binary.pensoft.net/file/1785603This dataset is made available under the Open Database License (http://opendatacommons.org/licenses/odbl/1.0/). The Open Database License (ODbL) is a license agreement intended to allow users to freely share, modify, and use this Dataset while maintaining this same freedom for others, provided that the original source and author(s) are credited. Han-Chi Lei, Jia Li, Tai-Jie Yu, Yu-Xian Gao, Zhong-Hua Wu, Yu-Cheng Dai, Tie-Zhi Liu, Run-Ke Mao, Wei-Jian Guan, Ying Tang, Ting Li, Yuan-Tian Lu, Qing-Wei Zang, Li-Ping Tang

Supplementary material 3 ITS fasta Data type fasta https://binary.pensoft.net/file/1785604This dataset is made available under the Open Database License (http://opendatacommons.org/licenses/odbl/1.0/). The Open Database License (ODbL) is a license agreement intended to allow users to freely share, modify, and use this Dataset while maintaining this same freedom for others, provided that the original source and author(s) are credited. Han-Chi Lei, Jia Li, Tai-Jie Yu, Yu-Xian Gao, Zhong-Hua Wu, Yu-Cheng Dai, Tie-Zhi Liu, Run-Ke Mao, Wei-Jian Guan, Ying Tang, Ting Li, Yuan-Tian Lu, Qing-Wei Zang, Li-Ping Tang

Supplementary material 4 LSU Data type fasta https://binary.pensoft.net/file/1785605This dataset is made available under the Open Database License (http://opendatacommons.org/licenses/odbl/1.0/). The Open Database License (ODbL) is a license agreement intended to allow users to freely share, modify, and use this Dataset while maintaining this same freedom for others, provided that the original source and author(s) are credited. Han-Chi Lei, Jia Li, Tai-Jie Yu, Yu-Xian Gao, Zhong-Hua Wu, Yu-Cheng Dai, Tie-Zhi Liu, Run-Ke Mao, Wei-Jian Guan, Ying Tang, Ting Li, Yuan-Tian Lu, Qing-Wei Zang, Li-Ping Tang

Supplementary material 5 tef1 fasta Data type fasta https://binary.pensoft.net/file/1785606This dataset is made available under the Open Database License (http://opendatacommons.org/licenses/odbl/1.0/). The Open Database License (ODbL) is a license agreement intended to allow users to freely share, modify, and use this Dataset while maintaining this same freedom for others, provided that the original source and author(s) are credited. Han-Chi Lei, Jia Li, Tai-Jie Yu, Yu-Xian Gao, Zhong-Hua Wu, Yu-Cheng Dai, Tie-Zhi Liu, Run-Ke Mao, Wei-Jian Guan, Ying Tang, Ting Li, Yuan-Tian Lu, Qing-Wei Zang, Li-Ping Tang

Supplementary material 6 rpb2 fasta Data type fasta https://binary.pensoft.net/file/1785607This dataset is made available under the Open Database License (http://opendatacommons.org/licenses/odbl/1.0/). The Open Database License (ODbL) is a license agreement intended to allow users to freely share, modify, and use this Dataset while maintaining this same freedom for others, provided that the original source and author(s) are credited. Han-Chi Lei, Jia Li, Tai-Jie Yu, Yu-Xian Gao, Zhong-Hua Wu, Yu-Cheng Dai, Tie-Zhi Liu, Run-Ke Mao, Wei-Jian Guan, Ying Tang, Ting Li, Yuan-Tian Lu, Qing-Wei Zang, Li-Ping Tang
