# Supplementary material for: Update and taxonomic revision of Ganoderma (Basidiomycota, Ganodermataceae) from China
Source: MycoKeys. 2026 Sep 21;140:261–97. doi: 10.3897/mycokeys.140.204190 (PMC13620809; doi:10.3897/mycokeys.140.204190)
Supplement: Supplementary file 1 [file mycokeys-140-261-s001.pdf]

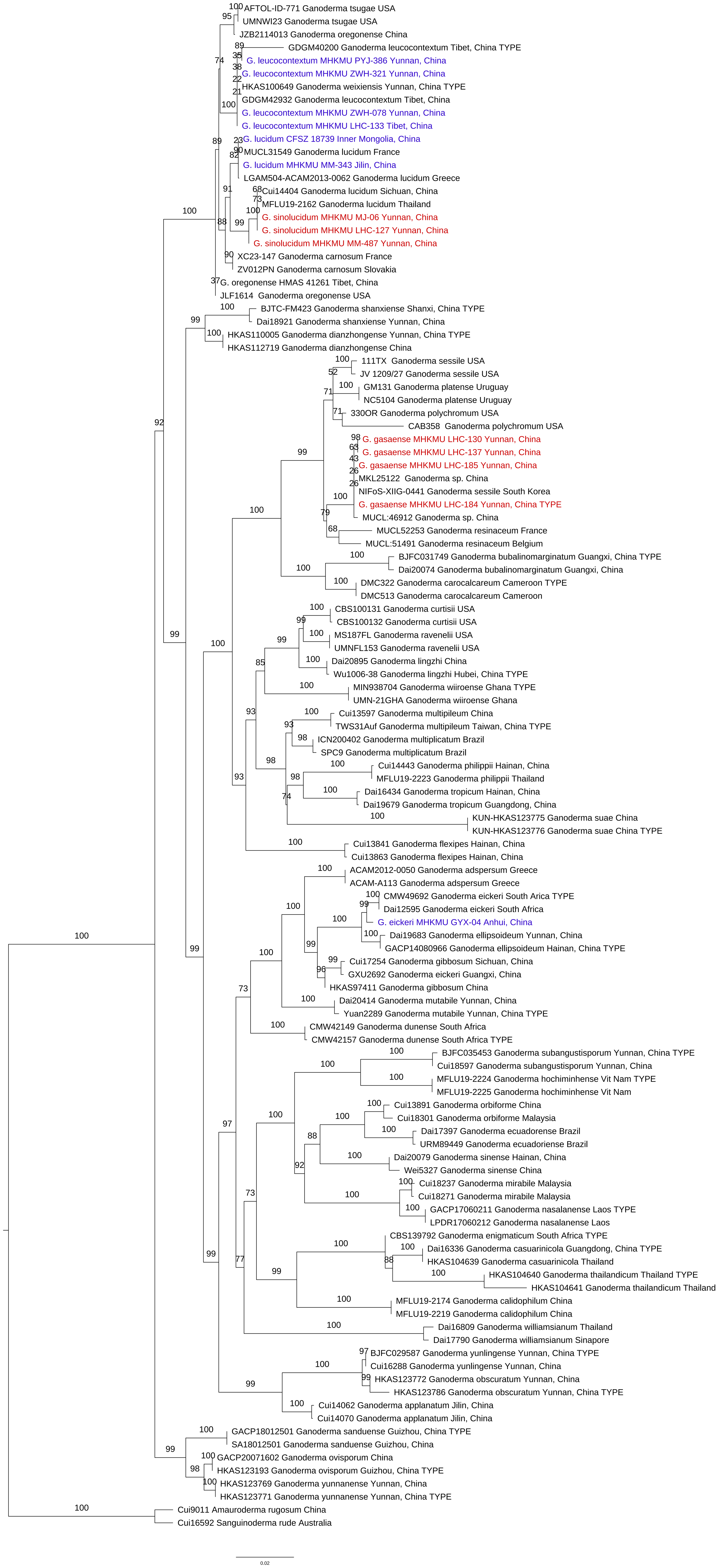
AFTOL-ID-771 Ganoderma tsugae USA
UMNWI23 Ganoderma tsugae USA
JZB2114013 Ganoderma oregonense China
GDGM40200 Ganoderma leucocontextum Tibet, China TYPE
G. leucocontextum MHKMU PYJ-386 Yunnan, China
G. leucocontextum MHKMU ZWH-321 Yunnan, China
HKAS100649 Ganoderma weixiensis Yunnan, China TYPE
GDGM42932 Ganoderma leucocontextum Tibet, China
G. leucocontextum MHKMU ZWH-078 Yunnan, China
G. leucocontextum MHKMU LHC-133 Tibet, China
G. lucidum CFSZ 18739 Inner Mongolia, China
MUCL31549 Ganoderma lucidum France
G. lucidum MHKMU MM-343 Jilin, China
LGAM504-ACAM2013-0062 Ganoderma lucidum Greece
Cui14404 Ganoderma lucidum Sichuan, China
MFLU19-2162 Ganoderma lucidum Thailand
G. sinolucidum MHKMU MJ-06 Yunnan, China
G. sinolucidum MHKMU LHC-127 Yunnan, China
G. sinolucidum MHKMU MM-487 Yunnan, China
XC23-147 Ganoderma carnosum France
ZV012PN Ganoderma carnosum Slovakia
G. oregonense HMAS 41261 Tibet, China
JLF1614 Ganoderma oregonense USA
BJTC-FM423 Ganoderma shanxiense Shanxi, China TYPE
Dai18921 Ganoderma shanxiense Yunnan, China
HKAS110005 Ganoderma dianzhongense Yunnan, China TYPE
HKAS112719 Ganoderma dianzhongense China
111TX Ganoderma sessile USA
JV 1209/27 Ganoderma sessile USA
GM131 Ganoderma platense Uruguay
NC5104 Ganoderma platense Uruguay
330OR Ganoderma polychromum USA
CAB358 Ganoderma polychromum USA
G. gasaense MHKMU LHC-130 Yunnan, China
G. gasaense MHKMU LHC-137 Yunnan, China
G. gasaense MHKMU LHC-185 Yunnan, China
MKL25122 Ganoderma sp. China
NIFoS-XIIG-0441 Ganoderma sessile South Korea
G. gasaense MHKMU LHC-184 Yunnan, China TYPE
MUCL:46912 Ganoderma sp. China
MUCL52253 Ganoderma resinaceum France
MUCL:51491 Ganoderma resinaceum Belgium
BJFC031749 Ganoderma bubalinomarginatum Guangxi, China TYPE
Dai20074 Ganoderma bubalinomarginatum Guangxi, China
DMC322 Ganoderma carocalcareum Cameroon TYPE
DMC513 Ganoderma carocalcareum Cameroon
CBS100131 Ganoderma curtisii USA
CBS100132 Ganoderma curtisii USA
MS187FL Ganoderma ravenelii USA
UMNFL153 Ganoderma ravenelii USA
Dai20895 Ganoderma lingzhi China
Wu1006-38 Ganoderma lingzhi Hubei, China TYPE
MIN938704 Ganoderma wiiroense Ghana TYPE
UMN-21GHA Ganoderma wiiroense Ghana
Cui13597 Ganoderma multipileum China
TWS31Auf Ganoderma multipileum Taiwan, China TYPE
ICN200402 Ganoderma multiplicatum Brazil
SPC9 Ganoderma multiplicatum Brazil
Cui14443 Ganoderma philippii Hainan, China
MFLU19-2223 Ganoderma philippii Thailand
Dai16434 Ganoderma tropicum Hainan, China
Dai19679 Ganoderma tropicum Guangdong, China
KUN-HKAS123775 Ganoderma suae China
KUN-HKAS123776 Ganoderma suae China TYPE
Cui13841 Ganoderma flexipes Hainan, China
Cui13863 Ganoderma flexipes Hainan, China
ACAM2012-0050 Ganoderma adspersum Greece
ACAM-A113 Ganoderma adspersum Greece
CMW49692 Ganoderma eickeri South Arica TYPE
Dai12595 Ganoderma eickeri South Africa
G. eickeri MHKMU GYX-04 Anhui, China
Dai19683 Ganoderma ellipsoideum Yunnan, China
GACP14080966 Ganoderma ellipsoideum Hainan, China TYPE
Cui17254 Ganoderma gibbosum Sichuan, China
GXU2692 Ganoderma eickeri Guangxi, China
HKAS97411 Ganoderma gibbosum China
Dai20414 Ganoderma mutabile Yunnan, China
Yuan2289 Ganoderma mutabile Yunnan, China TYPE
CMW42149 Ganoderma dunense South Africa
CMW42157 Ganoderma dunense South Africa TYPE
BJFC035453 Ganoderma subangustisporum Yunnan, China TYPE
Cui18597 Ganoderma subangustisporum Yunnan, China
MFLU19-2224 Ganoderma hochiminhense Vit Nam TYPE
MFLU19-2225 Ganoderma hochiminhense Vit Nam
Cui13891 Ganoderma orbiforme China
Cui18301 Ganoderma orbiforme Malaysia
Dai17397 Ganoderma ecuadorense Brazil
URM89449 Ganoderma ecuadoriense Brazil
Dai20079 Ganoderma sinense Hainan, China
Wei5327 Ganoderma sinense China
Cui18237 Ganoderma mirabile Malaysia
Cui18271 Ganoderma mirabile Malaysia
GACP17060211 Ganoderma nasalanense Laos TYPE
LPDR17060212 Ganoderma nasalanense Laos
CBS139792 Ganoderma enigmaticum South Africa TYPE
Dai16336 Ganoderma casuarinicola Guangdong, China TYPE
HKAS104639 Ganoderma casuarinicola Thailand
HKAS104640 Ganoderma thailandicum Thailand TYPE
HKAS104641 Ganoderma thailandicum Thailand
MFLU19-2174 Ganoderma calidophilum China
MFLU19-2219 Ganoderma calidophilum China
Dai16809 Ganoderma williamsianum Thailand
Dai17790 Ganoderma williamsianum Sinapore
BJFC029587 Ganoderma yunlingense Yunnan, China TYPE
Cui16288 Ganoderma yunlingense Yunnan, China
HKAS123772 Ganoderma obscuratum Yunnan, China
HKAS123786 Ganoderma obscuratum Yunnan, China TYPE
Cui14062 Ganoderma applanatum Jilin, China
Cui14070 Ganoderma applanatum Jilin, China
GACP18012501 Ganoderma sanduense Guizhou, China TYPE
SA18012501 Ganoderma sanduense Guizhou, China
GACP20071602 Ganoderma ovisporum China
HKAS123193 Ganoderma ovisporum Guizhou, China TYPE
HKAS123769 Ganoderma yunnanense Yunnan, China
HKAS123771 Ganoderma yunnanense Yunnan, China TYPE
Cui9011 Amauroderma rugosum China
Cui16592 Sanguinoderma rude Australia
0.02
